# A Complete Grocery Pick-and-Pack Application Using a Computationally Lightweight Vision-Based Mobile Manipulator

**DOI:** 10.3390/s26092860

**Published:** 2026-05-03

**Authors:** Thanavin Mansakul, Gilbert Tang, Phil Webb, Jamie Rice, Daniel Oakley, James Fowler

**Affiliations:** Centre for Robotics and Assembly, Faculty of Engineering and Applied Sciences, Cranfield University, Bedford MK43 0AL, UK

**Keywords:** vision-based grasping system, end-to-end grasp detection, mobile manipulator, lightweight computation, object detection, object pose estimation, machine vision

## Abstract

Mobile manipulators have become essential platforms for autonomous tasks that demand high-quality performance and efficient operational processes. This paper presents a complete grocery pick-and-pack system for a mobile manipulator, integrating a graphical user interface (GUI) with an end-to-end vision-based grasp detection pipeline designed for lightweight computation. The system is evaluated on the Grocery Pick-and-Pack Benchmark (Level-3), the most challenging level due to deformable objects, dimensional constraints, and strict grasp-point requirements. Experimental results demonstrate an average success rate of 92% across five item classes, with the deformable sweet bag the most challenging at 60% and an average execution time of 7.5 s on an edge device. The system achieves strong computational efficiency, reflected by a compute-to-speed ratio (CSR) of 0.008, with a total model size of only 30.9 MB. Performance is further validated across multiple hardware platforms and under real competition scenarios in the European Robotics League 2025. The findings highlight the practical impact of lightweight, vision-based mobile manipulation and provide insights into current challenges and future research directions for autonomous robotic applications.

## 1. Introduction

As many countries transition toward ageing societies [1], the development of smart cities has become increasingly important for enhancing quality of life and overall convenience. One key sector undergoing transformation is grocery retail [2]. Grocery shopping is an essential part of daily living, and the rise of click-and-collect and home-delivery services has intensified the need for efficient order-fulfilment systems. Traditionally, retailers rely on human workers to pick items from a customer’s order list, place them into baskets, and transport them to collection points or delivery vehicles.

However, this classical workflow faces several challenges arising from human error, such as selecting incorrect items, unpredictable processing times, and variability in workforce availability. Recent declines in birth rates have further contributed to labour shortages, while rising wages continue to increase operational costs. Research also highlights the growing labour gap and emphasises how artificial intelligence and emerging technologies can help address these issues [3]. Moreover, manual picking is prone to inaccuracies and delays, which can negatively affect customer satisfaction and reduce profitability for retailers. Autonomy offers a promising solution by reducing repetitive workloads and improving consistency. Importantly, such systems are not intended to replace human labour entirely. Instead, they complement human workers, who can shift toward higher-level responsibilities such as inspection, stock management, decision-making, and problem-solving, tasks that remain challenging for robots but are natural for humans.

To address the challenges outlined above, it is essential to analyse the traditional workflow and identify the key steps where advanced technologies can provide meaningful support. Since the process involves repeated pick-and-place actions and transportation of items across different locations, the combination of a robotic manipulator and a mobile platform represents an ideal solution. For the robot to select items according to the customer’s order list, reliable object detection and classification are required, making machine vision a critical component of the system.

Moreover, because the platform must operate autonomously and remain compact, lightweight computation becomes a priority. Efficient processing, reduced hardware size, and improved cost and energy performance are all necessary to ensure practical deployment. These requirements introduce several research challenges, particularly when aiming for robust performance in real-world environments.

The following section reviews related work on mobile manipulators and machine vision-based systems designed for similar tasks. This provides a foundation for identifying research gaps and opportunities for improvement in the context of autonomous grocery pick-and-pack operations.

### 1.1. Contributions

The present work builds directly on [4], which introduced the foundational grasp-point detection pipeline and validated it at Benchmark Levels 1 and 2 using geometrically simple objects under controlled laboratory conditions. The current paper extends that system in four technically distinct ways as shown in Table 1. First, it integrates the Level-3 benchmark scenario, involving deformable objects (sweet bag, conditioner), objects requiring non-centroid grasp points (cleaner spray, Spam can, tape), and strict dimensional constraints, none of which were evaluated in [4]. Second, it introduces the two-stage learning-based grasp-point detection module (Section 2.5), which is entirely absent from [4]; that work relied exclusively on the classical PCA-based method. Third, the system is evaluated in the European Robotics League 2025, a live competition environment that introduces hardware faults, unstable networking, and real-world task penalties not present in the controlled settings. Fourth, a cross-platform evaluation methodology (Section 2.8 and Section 3.3) is introduced and applied across four hardware configurations with standardised energy and cost efficiency metrics.

A direct quantitative comparison with the [4] system on identical test conditions was not performed for the following reason: the Level-3 benchmark items and the two-stage detection module did not exist in [4], making a like-for-like comparison impossible. The [4] system would fail entirely on Level-3 objects requiring learned grasp points, so reporting its score alongside the current system would not constitute a meaningful comparison. Instead, the present work treats [4] as the baseline design from which the new contributions depart, and documents the performance improvements afforded by those contributions.

The Grocery Pick-and-Pack Benchmark, defined in [4], structures the task into three levels of increasing difficulty. Level 1 targets geometrically simple objects with no shape complexity. Level 2 introduces moderate shape variation and object poses. Level-3, the focus of this paper, is the most challenging level: it includes deformable objects (a conditioner bottle and a sweet bag), objects requiring grasping at specific non-centroid locations (a cleaner spray bottle, a Spam tin, and a tape roll), and strict dimensional constraints arising from a 15-cm gripper width limit. The system must correctly classify each item, estimate object pose and its grasp point, and execute a pick-and-place routine without damaging the object and furniture, exceeding the gripper’s range, or dropping the item during the task.

This research provides the following key contributions:

1. End-to-end grocery pick-and-pack system. We present a complete vision-based mobile-manipulator system integrating grasp-point detection, motion planning, and a GUI into a seamless autonomous workflow. The system architecture (Section 2.1) branches between a classical method for geometrically regular items and a learning-based method for items requiring task-specific grasp points (Section 2.3, Section 2.4 and Section 2.5). Validated on the Level-3 Grocery Pick-and-Pack Benchmark (Section 3.1), the system achieved a 92% average success rate and a 7.5 s average pick time on an edge device. The system also ran on Episode 4 of the European Robotics League 2025 (Section 3.2), scoring 17 out of 36 points, which was well-recorded.
2. Computationally lightweight and portable inference framework. We present a compact inference stack with a total model size of 30.9 MB, comprising YOLOv11-Nano for object detection, FastSAM-s for instance segmentation, and a PCA-based grasp-pose estimator. The stack is implemented in Python 3.12 using PyTorch 2.0 and the Ultralytics and OpenCV libraries, and has been validated on three heterogeneous platforms: a performance laptop (Dell G16) with and without GPU, a compact laptop (Dell XPS 13), and an embedded AI device (Jetson Orin Nano). Implementation parameters sufficient for replication, including model variants, input resolutions, training configurations, and inference settings, are reported in Section 2.2, Section 2.3, Section 2.4 and Section 2.5.
3. Cross-device evaluation methodology. We propose a validation strategy comparing processing speed, energy efficiency, and cost across four hardware configurations (Section 3.3). The methodology defines the compute-to-speed ratio (CSR) and energy efficiency metric ($\eta_{E}$) introduced in Section 2.8, providing an objective basis for deployment trade-off decisions applicable to any mobile-manipulator system.

The remainder of this paper is organised as follows. Section 2 describes the system architecture and methodology. Section 3 reports experimental results on the Level-3 benchmark, the ERL 2025 competition, and across computing platforms. Section 4 discusses limitations and future directions. Section 5 concludes.

### 1.2. Related Work

#### 1.2.1. Mobile Manipulators for Autonomous Pick-and-Place

Mobile manipulators combine the flexibility of a mobile base with the dexterity of a robotic arm, making them a natural platform for autonomous retrieval tasks in dynamic, unstructured environments [5,6]. Early work demonstrated feasibility in semi-structured manufacturing cells [5,7], and subsequent studies extended these capabilities to broader Industry 4.0 settings [6]. A persistent limitation across these systems, however, is their dependence on pre-mapped environments and fixed object positions, assumptions that are violated in real retail environments where product placement varies, shelf clutter is common, and object pose cannot be predetermined.

In grocery-specific contexts, Bajracharya et al. [8] demonstrated end-to-end mobile manipulation on retail shelves and established a metrics-driven evaluation protocol that provides a useful precedent for quantitative benchmarking. Their system, however, required substantial onboard computing and did not treat computational footprint as a first-class design objective. Similarly, Shahria et al. [9] achieved high pick success rates in activities-of-daily living tasks but relied on a distance-based control algorithm and 3D coordinates using a depth frame, limiting deployment with respect to computational efficiency, user interface design, and practical use cases. The present work addresses both gaps: the system operates entirely on an edge device without image depth dependency (Section 2.1) and is validated under a standardised, publicly described benchmark with replicable performance metrics (Section 2.8).

#### 1.2.2. Vision-Based Grasping: Perception, Segmentation, and Grasp-Pose Estimation

Vision-based robotic grasping has advanced significantly through learning-based methods, yet no single approach generalises robustly across all object types and scene conditions. Du et al. [10] provide a structured taxonomy spanning object localisation, pose estimation, and grasp-point prediction, and note that most existing systems treat each stage independently, a design that introduces compounding errors and redundant computation between stages. This motivated the unified, end-to-end system architecture in the present work, which chains all stages into a single inference pipeline with shared intermediate representations.

Dong and Zhang [11] reviewed end-to-end grasp-detection systems and found that hybrid architectures, combining learning-based perception with classical geometric reasoning, outperform purely learning-based or purely classical approaches in cluttered scenes, particularly when training data are scarce. This finding directly informed our design choice: a classical method (PCA) handles geometrically regular items [12], while a learning-based method (YOLOv11-Nano) handles items that require task-specific grasp points not derivable from shape alone (Section 2.5). The use of YOLO-family architectures for grasping is a growing trend: Cong et al. [13] demonstrated real-time grasp-point detection in an industrial setting, confirming competitive speed and accuracy at low hardware cost. Compared with their system, the present work achieves a smaller model footprint (30.9 MB total) while maintaining a success rate above 90% across five items on the Level-3 benchmark.

For segmentation, FastSAM [14] offers a compelling balance between segmentation quality and computational cost compared with the full Segment Anything Model (SAM) or Mask R-CNN-based approaches. FastSAM runs inference in real time on edge devices without requiring class-specific training, aligning with the computationally lightweight design objective of this work. One identified limitation of the current pipeline is that object detection (YOLOv11) and segmentation (FastSAM) run as separate sequential modules for geometric items. Future work should investigate unified instance-segmentation-with-classification models that could eliminate this redundancy and reduce the total pipeline latency. In adjacent domains, vision-guided agricultural robots, such as those developed for apple harvesting [15], face analogous perception challenges: separating target objects from cluttered backgrounds and estimating approach vectors under shape and pose uncertainty. The key difference in the grocery context is that shelf items consistently face the camera, a constraint this system exploits by using RGB-only input rather than dense 3D point clouds, avoiding the substantial computational overhead associated with depth processing (Section 2.2).

#### 1.2.3. Lightweight Computation for Real-Time Robotic Perception

The majority of published vision pipelines for robotic manipulation are developed and benchmarked on high performance devices, making direct deployment on resource-constrained edge devices non-trivial without significant architecture redesign [16]. Zheng et al. [16] identify three concurrent requirements for practical edge deployment: near-real-time inference on limited hardware, sufficient accuracy to support reliable grasping, and a small enough model to permit onboard storage and rapid transfer. These three requirements are seldom all addressed simultaneously in the literature, and the trade-offs between them are rarely quantified across multiple hardware platforms.

The YOLO model family has emerged as the dominant solution for the speed–accuracy trade-off in object detection [17]. Jegham et al. [17] provide a comprehensive cross-version benchmark confirming that YOLOv11 variants define a new frontier across speed, accuracy, and model size, outperforming YOLOv8, YOLOv9, and YOLOv12 on standard detection benchmarks. The present work extends this finding to the domain of grasp-point detection: our experiments (Section 2.5) show that YOLOv11-Nano learns sub-object grasp regions from as few as 225 training images, substantially less data than required by dedicated grasp-detection networks such as GraspNet, without sacrificing real-time performance. Our own attempts to deploy GraspNet on this task (Section 4) confirmed that current dedicated grasp-estimation architectures are computationally heavier and less adaptable to novel object classes, motivating the use of a general-purpose detector fine-tuned for grasp-point localisation.

## 2. Proposed Methodology

Section 2 presents the proposed system in the order in which it is deployed. Section 2.1 describes the overall architecture and hardware communication. Section 2.2, Section 2.3, Section 2.4 and Section 2.5 detail the vision pipeline, object detection, segmentation, classical grasp-pose estimation for geometric items, and learning-based grasp-point detection for task-specific items, which together enable Contributions 1 and 2.

### 2.1. System Architecture

The proposed system demonstrates a complete autonomous grocery pick-and-pack workflow using an end-to-end, computationally lightweight grasp-detection pipeline deployed on a mobile manipulator. The system architecture incorporates two grasp-planning strategies based on object shape complexity. For simple, geometrically regular items, the pipeline relies on object detection followed by segmentation, traditional image-processing techniques, and principal component analysis to determine suitable grasping points. In contrast, objects with irregular geometries or those requiring grasping at a specific, non-centroid location are handled using a two-stage object-detection approach. This method enables the model to learn task-specific grasp points that classical algorithms cannot reliably infer.

**Figure 1:**
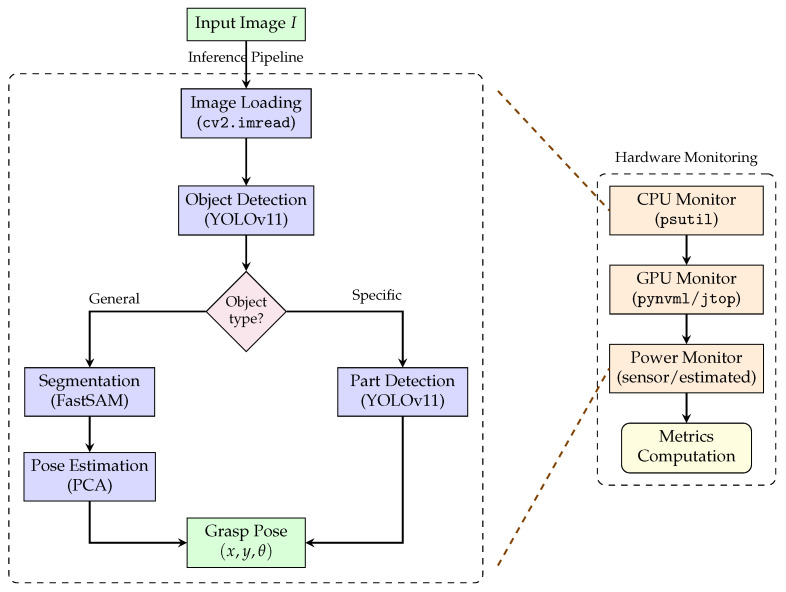
The evaluation system architecture employs an inference pipeline that first processes input images using YOLOv11 for object detection. The pipeline then branches according to object type: general objects are handled through FastSAM segmentation followed by PCA-based pose estimation, whereas specific objects (Cleaner Spray, Spam, Tape) are processed using a secondary YOLOv11 model for part-level detection. Both branches subsequently converge to generate the final grasp pose. Hardware monitoring modules operate concurrently as parallel threads.

The second detection stage overcomes the limitations of traditional vision-based grasping methods when applied to complex and diverse items. As a result, learning-based approaches are integrated to address these challenges and ensure robust performance in real-world environments. Figure 1 illustrates the overall system architecture, the algorithms employed in each module, and the hardware-level monitoring used to evaluate system performance.

This design ensures an end-to-end process with a strong focus on lightweight computation. For general objects, no prior knowledge or large training datasets are required. Through systematic evaluation and real-world experimentation, an image-processing pipeline was developed using statistical techniques, specifically Principal Component Analysis (PCA), to analyse pixel-level information. This reduces unnecessary data and dimensionality while preserving the essential features needed to determine grasping points. For objects requiring more specific grasp-affordance cues, the inputs, parameters, and augmentation strategies were further optimised to maximise the capability of the YOLO model. These optimisations enable the system to achieve accurate, robust grasp-point prediction with minimal training data and reduced training time, making it suitable for real-world grocery pick-and-pack applications. Finally, the object pose is estimated, and the corresponding robot motion plan is generated based on the pose information. Details of each module are presented in the following section.

**Figure 2:**
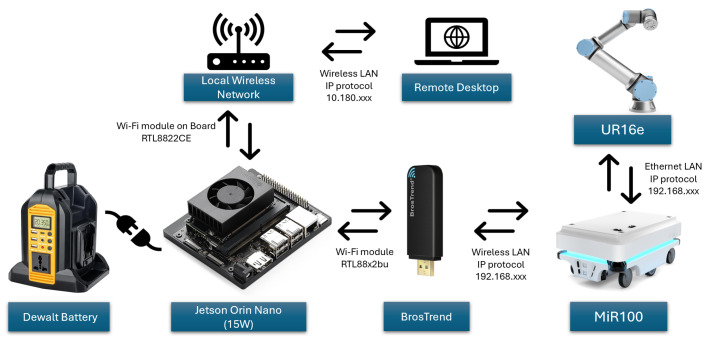
Hardware and communication architecture [4].

The hardware and communication architecture is illustrated in Figure 2. Communication between the remote laptop and the Jetson Orin Nano is wireless by design, as the Jetson is physically mounted on the MiR100 and must move with it; a tethered connection to the laptop would restrict the robot’s operational range. The Jetson-to-UR16e communication, by contrast, uses a wired Ethernet link (IP protocol, 192.168.x.x subnet), which was selected because the UR16e and the MiR100 are co-located on the same mobile platform and a physical cable of fixed length does not restrict mobility in this sub-system. The Jetson-to-MiR100 connection uses the MiR100’s onboard Wi-Fi module, because the MiR100 does not expose a standard Ethernet port for external device control in this configuration; its native control interface operates over IP and is accessible via the internal wireless network.

The primary limitation of this architecture is that Wi-Fi connections are inherently slower and less deterministic than wired Ethernet. In the laboratory setting and at ERL 2025, this did not cause control failures, as the Python-based RTDE control interface operates over TCP and tolerates occasional packet delay. However, in environments with high Wi-Fi congestion, a USB-to-Ethernet adapter attached to the Jetson and connected directly to the MiR100 via a short cable would be a more reliable alternative and is recommended for future deployments.

**Figure 3:**
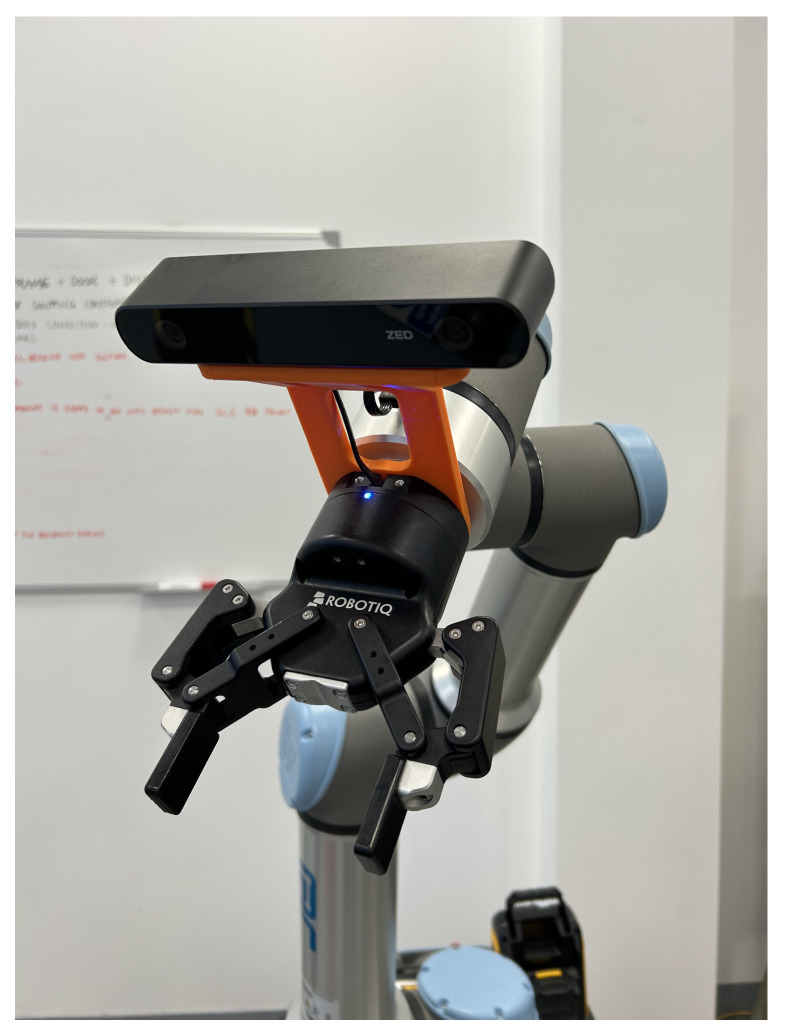
Eye-in-hand ZED 2i stereo camera paired with a Robotiq 2F-85 gripper.

At the end-effector, the perception and manipulation hardware consists of a ZED 2i stereo camera mounted in an eye-in-hand configuration and wired to the Jetson via USB, together with a Robotiq 2F-85 two-finger gripper attached to the UR16e flange, as shown in Figure 3. This arrangement allows the camera to move with the wrist during approach, providing viewpoints tailored to each target object, while the gripper executes the final grasp guided by the vision pipeline.

### 2.2. Object Detection

To achieve lightweight computation, several object-detection methods were evaluated, and YOLOv11 was selected for this project. As a current state-of-the-art model [17], it provides high processing speed, strong accuracy, and an open-source framework that can be adapted for task-specific requirements. This study includes detailed comparisons across multiple training configurations, including different input resolutions, image-augmentation strategies, and YOLO model variants. The results reveal a consistent trend: higher image resolutions increase computational burden while improving classification confidence. Consequently, selecting an appropriate resolution requires balancing accuracy with real-time performance.

For data augmentation, horizontal flips, brightness adjustments (−20% to +20%), and cropping (0–20% zoom) were applied to generate diverse training samples from the original dataset. These augmentations enhance the model’s robustness to real-world variability while keeping the training process efficient. A “resize-with-padding” strategy was also employed to convert all images into square dimensions, with unused regions filled in black. This approach simplifies subsequent segmentation by providing uniform background areas for contour extraction.

Among the evaluated architectures, the YOLOv11-Medium model achieved the highest overall performance. However, because the confidence threshold in this application is capped at 0.7, the YOLOv11-Nano model becomes the most suitable choice due to its superior speed and significantly smaller size. Larger models do not provide meaningful improvements under these constraints, underscoring the importance of aligning model selection with application-specific requirements and empirical validation.

The YOLOv11-Nano model was trained using the Ultralytics framework. Training configuration: epochs = 100, batch = auto (GPU memory-adaptive), input resolution = 640 × 640, optimiser = AdamW (lr = 0.001, weight decay = 0.0005), confidence threshold = 0.70, IoU threshold = 0.45. The model was trained for 100 epochs on a single NVIDIA GPU.

The target application also influences the choice of sensing modality. Grocery items are typically arranged facing outward on shelves, allowing the robot to capture clear frontal images that provide the most informative viewpoint for recognition. In this context, generating a full point cloud is unnecessary and may even be counterproductive, as it introduces background clutter and neighbouring objects that are irrelevant to the task. Since the objective is to identify and retrieve only the items listed in the shopping order, RGB imagery is the most appropriate input modality. It directly supports object detection while avoiding the computational overhead associated with dense 3D data. Moreover, relying solely on RGB images contributes to lightweight computation by ensuring that only essential visual information is processed.

RGB images are widely used in vision-based systems because they provide rich visual information comparable to human perception. Image resolution is a critical factor in producing sharp and realistic representations; however, higher resolutions require substantially more storage and computational resources. A common strategy for reducing computational load is therefore to use the lowest feasible resolution, which decreases processing time and memory usage. The trade-off is that low-resolution images may appear blurred or lack detail, potentially affecting downstream tasks.

Identifying an optimal resolution that balances clarity and efficiency is essential. In this study, the results show that the lowest resolution available from the ZED 2i camera is sufficient for object detection, even though the associated confidence scores are lower than those obtained with higher-resolution inputs. While higher confidence is generally desirable, it also increases computational cost and may reduce the overall detection rate. For the grocery pick-and-pack application, a confidence threshold of 0.7 was selected, as it provides reliable classification and detection across diverse scenarios.

An RGB image alone may not provide the most comprehensive information for grasping, but this task benefits from the consistent presentation of items on grocery shelves, where products are typically oriented outward for customer visibility. In contrast, other studies aim to capture more complete object information through multi-view imaging, 3D reconstruction, or dense point-cloud estimation. These approaches can improve accuracy but introduce significant computational overhead.

Given the need for lightweight computation and real-time performance in this application, RGB imagery represents an appropriate and efficient input modality.

### 2.3. Segmentation

When moving to geometric objects, segmentation plays a crucial role in extracting object-level information from images. Operating at the pixel level, it generates precise object boundaries that are essential for analysing item geometry and estimating reliable grasping points. Multiple segmentation approaches exist, and the choice depends heavily on the intended application. In this work, lightweight computation and practical deployment were key requirements, making FAST-SAM [14] the most suitable option. It provides an efficient model with a compact footprint, fast inference, no training requirement, and accurate segmentation outputs.

Future improvements in segmentation algorithms that combine both segmentation and classification would be highly beneficial. Such an approach would eliminate the need for a separate object-detection module, reducing redundancy and simplifying the pipeline. At present, segmentation remains an essential step for the grocery pick-and-pack task because accurate detection and classification must be complemented by precise object-shape extraction. This is particularly important for geometric-shape items, where centroid estimation contributes directly to grasp stability.

FastSAM-s was used without fine-tuning, as it operates as a class-agnostic segmentation model. The model was applied to the cropped bounding-box region returned by Stage 1 detection rather than the full image, reducing both inference area and computation. The FastSAM point-prompt mode was used: the centroid of the Stage 1 bounding box was passed as the foreground point prompt, and no background points were provided. Segmentation output is a binary mask of the same spatial dimensions as the cropped input; pixels belonging to the detected object are set to 1 and background pixels to 0.

### 2.4. Object Pose Estimation

In this module, which forms part of the sub-system for simple geometric-shape objects, the process begins after segmentation. To analyse the segmented region and determine a stable grasping point, principal component analysis (PCA) is employed as a statistical tool. PCA reduces the dimensionality of the pixel distribution and identifies the dominant directions of variation. In this context, the segmented object is represented by its x–y pixel coordinates, and PCA is used to determine the overall trend of the pixel distribution. The first principal component corresponds to the primary axis (interpreted as the object’s height), while the second principal component represents the secondary axis (interpreted as the object’s width or grasping side). Notably, PCA also provides the centroid of the segmented region, which serves as a suitable grasping point for ensuring grasp stability.

The binary mask from FastSAM is converted to a set of (x, y) pixel coordinates for all foreground pixels. PCA is applied to this 2D point set: the first principal component (PC1) corresponds to the axis of maximum variance and is interpreted as the object’s primary axis (height axis), while the second principal component (PC2) corresponds to the secondary axis and is the candidate grasping approach direction.

This method is highly efficient because it requires only binary pixels within the object boundary, without background or neighbouring objects, resulting in very low computational demand. Furthermore, PCA involves straightforward mathematical operations rather than complex neural-network inference, which contributes to fast and stable performance. However, image resolution influences both computation and output quality: higher-resolution images contain more pixels and therefore require more processing time. Additionally, the endpoints of the primary and secondary axes must be constrained within the object’s bounding box. Depending on the PCA output, these axes may be excessively long or short. To ensure proper visualisation and consistent interpretation, it is recommended to use the bounding-box information to extend or limit the axis lengths, as they follow linear equations.

The grasp orientation angle θ is defined as the direction of the second principal component (PC2), which represents the object’s minor axis and therefore the preferred closing direction for a parallel-jaw gripper: closing along the short axis yields stable two-line contact against the long body of the object. At execution time, the gripper’s tool frame is commanded to rotate about the world z-axis so that the finger-closing axis aligns with θ before descent.

Classical Grasp-Pose Estimation (for Geometric Objects) is shown below:

1. Input: RGB image; bounding box $B=[x_{\min},y_{\min},x_{\max},y_{\max}]$ from Stage 1 detection.
2. Instance segmentation(FAST-SAM): Use the FastSAM mask produced in Section 2.3 as the binary foreground mask.
3. Contour extraction: Apply cv2.findContours() to *M* to identify the largest connected foreground region *C*. Discard noise contours smaller than 100 pixels.
4. PCA: Extract all (x,y) pixel coordinates of foreground pixels in *C*. Apply PCA to the 2D point set using numpy.linalg.eigh on the covariance matrix. The eigenvector $v_{1}$ (larger eigenvalue) is the primary axis; $v_{2}$ is the secondary axis. The PCA centroid (mean of all foreground pixels) defines the grasp point $\left(x_{\mathrm{img}},y_{\mathrm{img}}\right)$. The orientation angle is computed as:
  $$\theta =\arctan2(v_{2}\left[1\right],\;v_{2}\left[0\right]).$$
5. Output: The algorithm returns the 2D grasp point $\left(x_{\mathrm{img}},y_{\mathrm{img}}\right)$ and the in-plane orientation angle θ. These values are subsequently forwarded to the depth-processing module to obtain the corresponding point-cloud coordinates (X,Y,Z) and to compute the roll component of the grasp pose.

The PCA step itself requires no training and runs on CPU using NumPy and OpenCV; it consumes the segmentation mask produced in Section 2.3. It is appropriate for objects with well-defined geometric centroids (e.g., bottles, bags), but cannot reliably identify non-centroid grasp affordances on objects with task-specific grip regions, motivating the learning-based path for those items.

### 2.5. Two-Step Grasping Point Detection

In this stage, we observed and evaluated several algorithms from object detection to grasp detection. This research found that the YOLOv11-nano model delivers excellent performance for grasp-point detection. During training, only a small grasping region within each object was annotated, yet the model successfully learned these features and predicted grasping points with high precision. Although YOLO-based architectures are typically used for object detection, our results demonstrate that they can also identify specialised sub-regions within an object, showing strong capability for fine-grained localisation. This contributes to both high speed and computational efficiency, making YOLOv11-nano an attractive choice, whereas dedicated grasp-prediction networks often provide grasp points, but do not prioritise lightweight computation.

**Figure 4:**
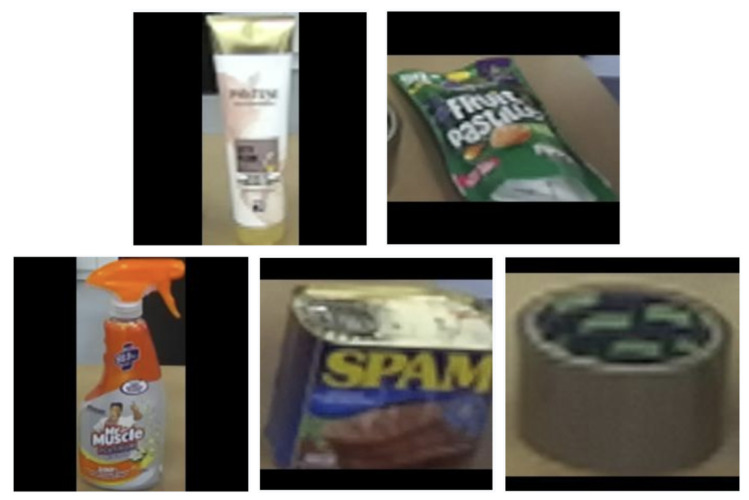
Preprocessing and augmentations.

Because lightweight computation is a core objective of this project, we further optimised the image-processing pipeline prior to model training. Smaller input images reduce computational load, but the resolution must remain sufficient to preserve essential features. By analysing the captured dataset, we identified the largest object, the conditioner bottle, as the limiting case, requiring a minimum resolution of 225 × 225 pixels to retain critical visual information. Reducing the image size below this threshold caused loss of detail and degraded detection performance. All other items were padded with black borders to match this target size, ensuring consistent input dimensions as shown in Figure 4. Otherwise, training directly on raw images would have forced the system to upscale to the maximum resolution, increasing computation and reducing real-world performance.

Training used the default configuration: epochs = 100, batch = −1 (auto mode for GPU), imgsz = 225, optimizer = auto (AdamW), and an example learning rate = 0.001. The training set comprised 225 images, including both raw and preprocessed variants. The dataset split was intentionally skewed toward training (90% train, 5% validation, 5% test) to prioritise rapid convergence and reduced wall-clock training time. Because the target deployment environment is highly constrained and contains no unseen object classes or novel backgrounds, the study did not prioritise explicit overfitting mitigation; the objective was to maximise accuracy for a routine, closed-world task.

With this optimised preprocessing strategy, the model required neither high-resolution images nor large quantities of training data. The grasping annotations were defined by bounding boxes around the graspable region, with the centre of each box used as the grasping point. For the tape item, the grasping points were defined as the centres of the bounding boxes on each side, capturing the essential grasp-affordance structure. This development approach enabled the system to achieve both high success rates and a lightweight model architecture. The Section 3 presents the detailed performance outcomes.

#### 2.5.1. Dataset and Training Details

Dataset for Stage 1 (Object Classification). The Stage 1 dataset covers all items in the Grocery Pick-and-Pack Benchmark Level-3: conditioner, sweet bag, cleaner spray, Spam can, and tape. Images were captured using the ZED 2i camera in the laboratory environment at a distance of approximately 0.5–1.0 m from the shelf, matching the operational distance of the robot. A total of 701 images were collected per class, with the following augmentations applied: horizontal flip (*p* = 0.5), brightness adjustment (−20% to +20%), and random crop (0–20% zoom-out). Images were resized with padding to 640 × 640 pixels. Dataset split: 80% training, 10% validation, 10% test.

Dataset for Stage 2 (Grasp-Point Detection). The Stage 2 dataset covers the three objects requiring task-specific grasp points: cleaner spray, Spam can, and tape. Images were captured from the same operational distance and viewpoint as the robot’s eye-in-hand camera. A total of 225 images were collected across the three classes, including both raw frames and versions pre-processed by the padding pipeline described in Section 2.5. Annotation was performed manually using a bounding-box labelling tool (Roboflow): for each image, a single bounding box was drawn around the graspable sub-region of the object (e.g., the neck of the cleaner spray, the lid of the Spam can, each flat face of the tape roll). The centre of this bounding box defines the grasp point used at inference time. For the tape item, two bounding boxes were annotated per image, one on each flat face of the roll. At inference time, the face with the higher YOLO confidence score was selected as the grasp target; if both scores were within 0.05 of each other, the face closer to the camera in the image plane (larger bounding-box area) was used. This heuristic handles cases where the tape is partly occluded or oriented at an angle to the camera. Dataset split: 90% training, 5% validation, 5% test, intentionally skewed toward training because the deployment environment is closed-world and the object set is fixed. Training configuration: epochs = 100, batch = auto, input resolution = 225 × 225, optimiser = AdamW (lr = 0.001).

The graspable sub-region was defined per object class as follows:

- Cleaner spray: The trigger handle, bounded by the top of the nozzle and the bottom of the handle grip.
- Spam can: The lid rim, bounded by the top edge of the can and extending 1 cm down the side.
- Tape: Each flat annular face, bounded by the outer edge and the central hole.

These regions were chosen because they (a) present two approximately parallel surfaces for the two-finger gripper to contact and (b) sit near the object’s centre of mass, minimising tip-over torque during lift.

Competition Dataset (ERL 2025). For the ERL 2025 episode, a new Stage 1 dataset was collected on-site covering the nine competition items: chocolate coins bag, Alpro custard carton, honey bottle, jelly tart, rice-cake pack, seed mix bag, pasta pack, water bottle, and Rubicon drink bottle. A total of 51 images were collected from multiple distances and viewing angles. Dataset split: 80/10/10 (train/val/test). Despite the small size, the model achieved reliable detection confidence above 0.7 on all items, demonstrating the system’s ability to adapt rapidly to new environments with minimal data collection.

#### 2.5.2. 3D Grasp-Point Estimation from RGB Detection Output

The vision pipeline (Section 2.2, Section 2.3, Section 2.4 and Section 2.5) operates on RGB images and produces a 2D grasp point $\left(x_{\mathrm{img}},y_{\mathrm{img}}\right)$ in pixel coordinates, along with an orientation angle θ. This 2D output is insufficient for robot motion planning, which requires a full 3D grasp pose in the robot base frame. The 2D-to-3D conversion proceeds as follows:

- Camera-frame 3D point cloud: Using the ZED 2i intrinsic calibration parameters (focal lengths $f_{x},f_{y}$ and principal point $c_{x},c_{y}$), the 3D camera-frame coordinates are computed as:
  $$X_{\mathrm{cam}}=\frac{\left(x_{\mathrm{img}}-c_{x}\right)\;Z_{\mathrm{cam}}}{f_{x}},\;Y_{\mathrm{cam}}=\frac{\left(y_{\mathrm{img}}-c_{y}\right)\;Z_{\mathrm{cam}}}{f_{y}}$$
- Robot-frame transformation: The camera-frame point is transformed to the robot base frame using the full kinematic chain
  $$T_{\mathrm{base}\to \mathrm{gripper}}\cdot T_{\mathrm{gripper}\to \mathrm{camera}}$$
  constructed from the robot’s current joint angles and the custom camera-mount dimensions (Section 2.7). This yields the 3D grasp pose $\left(X_{\mathrm{robot}},Y_{\mathrm{robot}},Z_{\mathrm{robot}},RX_{\mathrm{robot}},RY_{\mathrm{robot}},RZ_{\mathrm{robot}}\right)$ in the robot base frame.
- Motion command: This pose is passed to the UR16e’s RTDE controller as a target Cartesian waypoint. The robot’s onboard inverse kinematics solver computes the joint angles required to reach the pose.

This architecture decouples the detection and 3D estimation concerns: the RGB-based vision pipeline is optimised for fast, accurate 2D localisation, while the ZED 2i point cloud provides 3D point measurement needed for 3D conversion without burdening the neural network with depth processing. The key assumption is that the point cloud at the predicted grasp-point pixel is valid, an assumption that generally holds for the item surfaces in the benchmark but may fail for reflective or transparent objects, as discussed in Section 4.

Although the ZED 2i produces a calibrated depth map alongside each RGB frame, depth is deliberately excluded from the detection and segmentation modules and reserved for a single, well-defined step in the pipeline. Three considerations motivate this design. Grocery items on a shelf are consistently oriented toward the camera, so RGB alone carries sufficient information for classification; adding a depth channel would enlarge the model input without a matching gain in accuracy for this geometry. Stereo depth is also known to degrade on precisely the surfaces this benchmark contains: reflective metal tins produce specular highlights that corrupt stereo matching, transparent and semi-transparent containers such as the honey bottle in the ERL 2025 item set that scatter the disparity signal, and deformable items such as the sweet bag that present surface normals that shift with every grasp attempt, making depth map based shape analysis unreliable across trials. Feeding such measurements into detection or segmentation would inject noise at the most sensitive stage of the pipeline. Finally, carrying depth alongside RGB raises memory bandwidth and inference time, working directly against the lightweight-computation objective. RGB, by contrast, remains stable across all of these surface types provided the item is visible and within range, and is therefore adopted as the sole input modality for detection and grasp-point prediction.

Point cloud is instead applied at a single downstream step, once the 2D grasp point $\left(x_{image},y_{image}\right)$ has been predicted. The corresponding point cloud value is read from the ZED point cloud at that pixel through the ZED SDK (sl.point_cloud.get_value), lifting the image coordinate into a 3D camera-frame point $\left(x_{camera},y_{camera},z_{camera}\right)$ that is subsequently transformed into the robot base frame via the kinematic chain. Because point cloud is queried at one pixel rather than across the full image, the 3D information needed for motion planning is obtained at negligible computational cost, preserving the efficiency rationale of the RGB-only front end while still giving the manipulator an accurate target in Cartesian space.

### 2.6. Graphic User Interface (GUI)

To enable item selection from the shopping list, a graphical user interface (GUI) was developed to model each product and allow the operator to issue commands intuitively. Once an item is selected, the robot executes the corresponding pick-and-place routine until the object is successfully delivered or the attempt fails. The GUI also provides essential manual controls for the robotic arm, including emergency stop, Cartesian movements along axes, predefined poses such as home and hold, and real-time video feedback. Although the mobile base includes its own web-based interface, the custom GUI is particularly valuable when the robot behaves unexpectedly. Manual teleoperation complements autonomous operation and is fundamental for laboratory experimentation, debugging, and future system extensions, such as adding item metadata or reference images.

**Figure 5:**
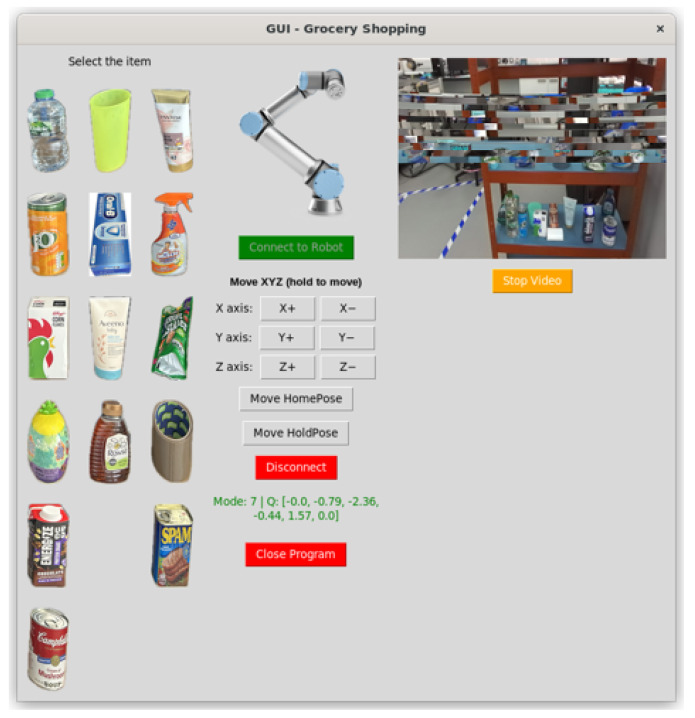
Graphical User Interface design for item selection, teleoperation, and live video.

The GUI in this project was implemented using Tkinter, a Python-based framework chosen for its rapid prototyping capability, cross-platform compatibility, simplicity for educational use, stability, and lightweight footprint [18,19]. The interface layout follows a grid-based structure, where each function is assigned to a specific row and column for clarity and maintainability. Each button, label, and image is associated with a dedicated callback function to ensure modularity and facilitate error handling. The application runs in a continuous loop, allowing each process to be validated, either successful or failed, before the system proceeds to the next step. The GUI is shown in Figure 5.

The pseudocode in Algorithm 1 presented below describes not only an application but a teleoperation design in which a laptop functions as the primary wireless controller; on-site interventions are reserved for emergency or safety cases requiring manual operation. In this implementation, the MiR100 mobile base remains controllable via its online application over Wi-Fi and IP, while the UR16e manipulator, previously operated exclusively with a time-consuming teach pendant, is now controllable through a custom Python GUI. The GUI exposes UR16e control primitives, integrates MiR100 functions, and orchestrates perception, planning, and execution into a single, seamless workflow. This architecture offers simplicity and portability, enabling rapid deployment on standard development hosts, and delivers responsive, robust teleoperation by using lightweight Python libraries and a clear concurrency model. The system’s concise, modular structure also facilitates debugging and extension, allowing new items, behaviours, or safety features to be added with minimal changes to the core codebase.
**Algorithm 1:** Logical workflow of the Graphical User Interface for the grocery pick-and-pack application
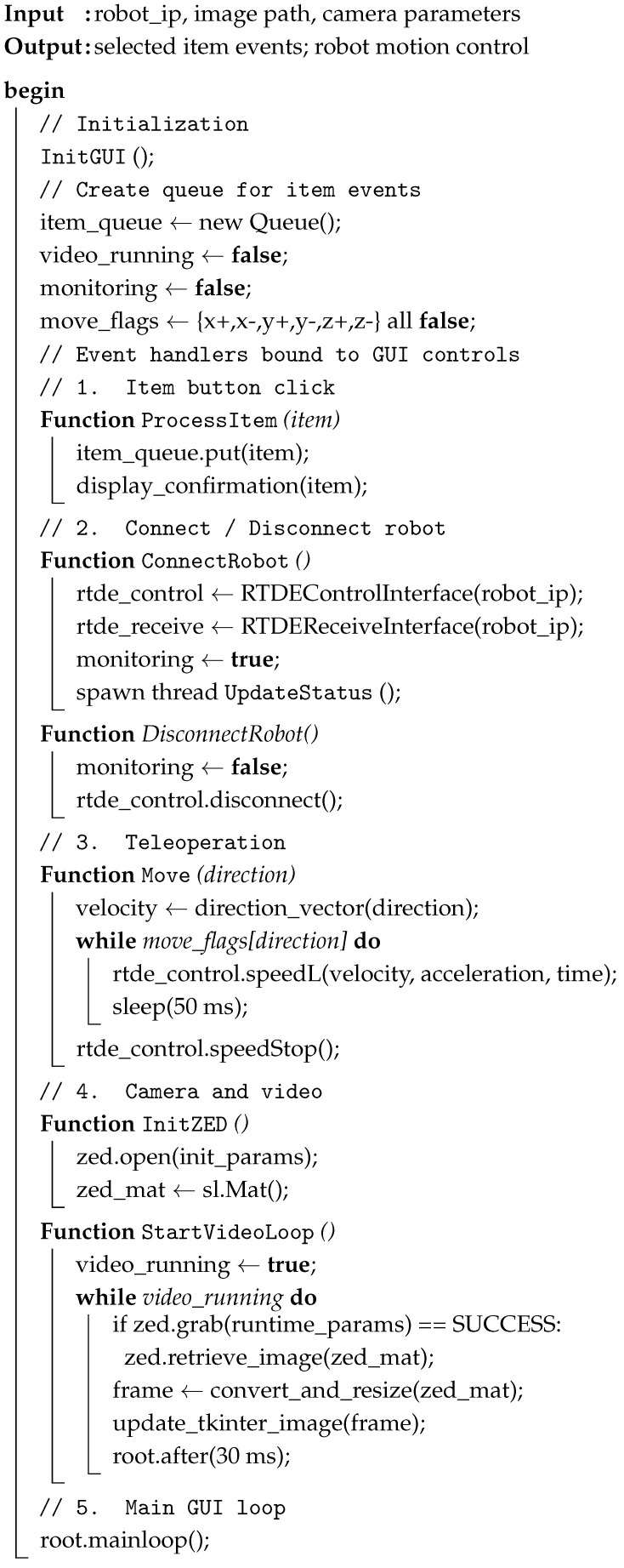


### 2.7. Robot Motion Planning and Control

This research highlights that the key to achieving reliable robot motion guided by machine vision is the development of an accurate kinematic model. Although the camera inherently contains factory-specified errors and tolerances, these can be minimised through recalibration. The camera mount was custom-designed and installed on the robot’s wrist, enabling the formation of a complete kinematic chain. The UR16e provided six revolute joints, and the additional components, the modified camera mount and the extended gripper, were modelled as homogeneous transformation matrices based on their custom dimensions. These matrices were then integrated into the main kinematic structure of the UR16e. The fundamental equations and modelling principles follow the methodology established in [4].

**Figure 6:**
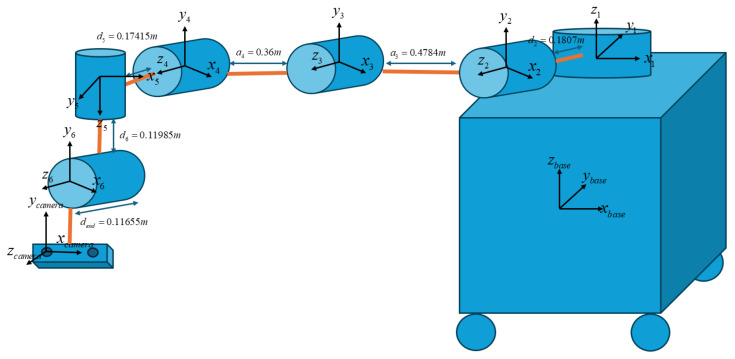
Home pose configuration for kinematic model identification [4].

A crucial requirement for constructing a kinematic model is that the robot must begin from its factory-defined home configuration. The coordinate frame at the robot base serves as the global reference for the entire system. All additional installations, such as the camera and gripper, are dimensioned relative to this base frame when the robot is in its home position. This ensures that the overall system is properly integrated and avoids ambiguity in axis directions, sign conventions, and coordinate alignment. Once the full kinematic chain is established, a fine-tuning process is performed. The example of home pose and dimension to find the kinematic model of this robot is shown below in Figure 6.

In practice, when the machine-vision module predicts a grasping point on the target object, that point is physically marked. The robot is then executed to reach the predicted location, and any deviation in each axis is measured and compensated through offset values. The robot can also perform this procedure with the gripper closed, allowing measurement of the distance between the gripper centre and the marked point. This technique is both simple and precise because it calibrates the entire system directly, reducing accumulated error across devices. For example, individual errors from the camera, robot, and gripper would otherwise compound, but the offset calibration effectively compensates for the combined system error.

This offset calibration is performed once per system configuration, after any hardware change to the camera mount, gripper, or tool flange, and compensates for the combined mechanical and optical misalignment between the camera-frame predictions and the robot’s commanded end-effector pose. The same offset applies to all objects and all grasps; it does not require per-object tuning, preserving the system’s ability to handle new items without recalibration.

### 2.8. Benchmark and Validation

**Figure 7:**
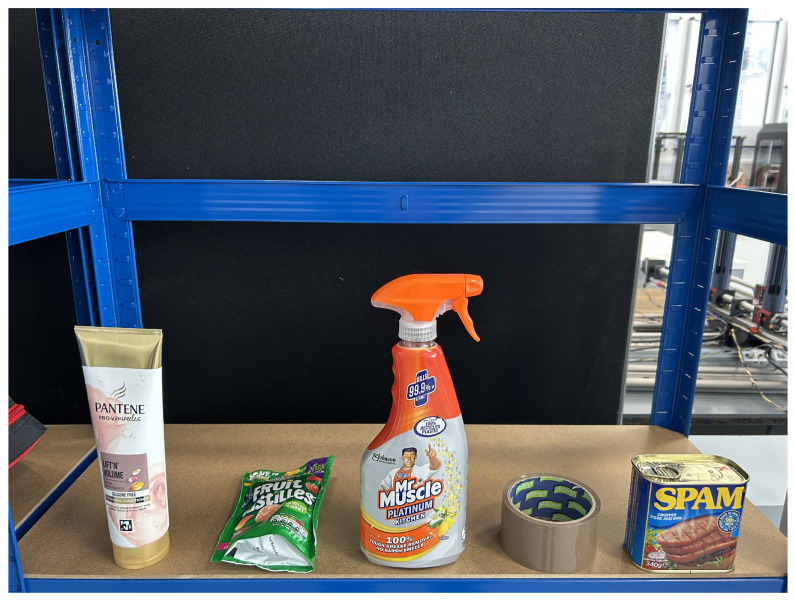
Item set for the Level-3 benchmark with random placement per trial [4].

Benchmark Level 3 Item Set, illustrated in Figure 7. The five items in the Level-3 benchmark are:

1. Conditioner (295 g, deformable squeeze bottle), grasped at the body using the classical method.
2. Sweet bag (147 g, flexible multi-layer film bag), grasped at the body using the classical method; its deformability and slippery surface make it the most failure-prone item.
3. Cleaner spray (481 g, rigid with a spray-trigger handle), where the trigger handle defines the required grasp point. This point cannot be reliably inferred from geometry alone, necessitating the learning-based method.
4. Tape (82 g, rigid cylindrical object with two valid grasp faces), grasped on either flat face, with the learning-based method predicting the face centre.
5. Spam (407 g, rigid rectangular can), grasped at the lid rim. The non-centroid grasp point again requires the learning-based method.

#### Definition of Success and Failure Criteria

A trial is counted as a success if the robot:

- Correctly detects and classifies the target item;
- Positions the gripper at the predicted grasp point within an acceptable positional tolerance;
- Closes the gripper on the object without dropping it during transport;
- Places the object in the designated delivery area.

A trial is counted as a failure if any of the following occur:

- The object is not detected;
- The grasp point is predicted incorrectly;
- The object slips from the gripper during transport;
- The object falls during placement.

To analyse the system across different devices, we use the computation-to-speed ratio in real experiments, as it requires real power energy to run the device. It can be measured through the power meter or battery station. In this case, the main device is the Jetson orin nano, so the used power can be shown in the battery station. In other cases, a power meter can show the energy consumption from using devices, such as personal computers, laptops, and edge devices. However, if we want to see the speed and energy consumption of the CPU and GPU in depth, we need power measurements on each platform to obtain insight into their performance and to conduct evaluation.

The compute-to-speed ratio (CSR) is introduced as a direct measure of the energy efficiency of the vision pipeline, defined as the ratio of processing throughput to instantaneous power draw:

(1) $$\mathrm{CSR}=\frac{\mathrm{FPS}}{\bar{P}},$$

where FPS is the frame rate sustained by the inference pipeline and $\bar{P}$ is the average power consumption in watts over the same interval. CSR expresses how many frames the system processes per watt consumed, which is a meaningful figure of merit for mobile robotic platforms where onboard energy is limited and sustained operation depends on managing the compute-to-power trade-off. A higher CSR indicates that the system delivers more useful visual processing per unit of energy, i.e., a more favourable balance between computational throughput and electrical cost.

Table 2 summarizes the measurement methods for each platform. Firstly, CPU Power Estimation is estimated using a linear interpolation model based on CPU utilisation and dynamic frequency scaling [20]:

(2) $$P_{\mathrm{CPU}}=P_{\mathrm{base}}+\alpha \cdot \left(P_{\mathrm{turbo}}-P_{\mathrm{base}}\right)$$

where $P_{\mathrm{base}}$ is the base Thermal Design Power (TDP), $P_{\mathrm{turbo}}$ is the maximum turbo power, and α∈[0,1] is the load factor computed as:

(3) $$\alpha =\frac{1}{2}\left(\frac{U_{\mathrm{CPU}}}{100}+\frac{f_{\mathrm{cur}}-f_{\mathrm{base}}}{f_{\max}-f_{\mathrm{base}}}\right)$$

where $U_{\mathrm{CPU}}$ is the CPU utilisation percentage obtained via psutil.cpu_percent(), $f_{\mathrm{cur}}$ is the current clock frequency from psutil.cpu_freq(), and $f_{\mathrm{base}}$, $f_{\max}$ are the base and maximum frequencies, respectively. Per-core utilization is also monitored using psutil.cpu_percent(percpu=True) to analyze workload distribution across processor cores.

The CPU power model in Equations (2) and (3) is formulated as a linear interpolation between the manufacturer-published base TDP ($P_{\mathrm{base}}$) and the maximum turbo power ($P_{\mathrm{turbo}}$), gated by a load factor α that combines instantaneous CPU utilisation and clock-frequency scaling. The model relies on two assumptions: (i) the relationship between utilisation, frequency, and package power is approximately linear within the normal operating envelope of the processor, and (ii) the chip is not thermally throttled, such that the current frequency $f_{\mathrm{cur}}$ reflects the load requested by the operating system rather than a thermal limit. When thermal throttling occurs, $f_{\mathrm{cur}}$ drops below the scheduler-commanded value, causing α to underestimate the true power and biasing the reported CPU power downward by a factor proportional to the throttle margin.

An external wall-plug power meter on a CPU-only platform was not validated; therefore, all CPU-derived power values are labelled as estimated and should be interpreted with a typical uncertainty of 10–25%, consistent with prior reports of similar load–frequency models. In contrast, direct sensor readings, NVML for the discrete GPU and INA3221 for the Jetson, are reported as direct and carry substantially lower uncertainty (typically <5%), inherited from the accuracy of the on-device sensors.

Secondly, using Discrete GPU Power Measurement (NVIDIA), power is measured directly via the NVIDIA Management Library (NVML) [21]:

(4) $$P_{\mathrm{GPU}}\left(t\right)=\mathrm{nvmlDeviceGetPowerUsage}\left(t\right)\times 10^{-3}\;\left[W\right]$$

Thirdly, for NVIDIA Jetson platforms, the INA3221 power monitor provides per-rail power measurements via the jetson-stats library [22]:

(5) $$P_{\mathrm{total}}=P_{\mathrm{GPU}}+P_{\mathrm{CPU}}+P_{\mathrm{SOC}}$$

where individual rail power values are obtained from jtop.power with measurements reported in milliwatts and converted to watts. The unified memory architecture of Jetson platforms means that memory power is included in the SOC measurement.

In terms of energy consumption, total energy consumption is computed by integrating instantaneous power over the inference duration:

(6) $$E=\int_{t_{0}}^{t_{f}}P\left(t\right)\;dt$$

For discrete sampling at interval δ, the energy is approximated as:

(7) $$E\approx \sum_{i=1}^{N}P\left(t_{i}\right)\cdot \delta =\bar{P}\cdot \Delta t$$

where *N* is the number of samples, $\bar{P}=\frac{1}{N}\sum_{i=1}^{N}P\left(t_{i}\right)$ is the average power, and $\Delta t=t_{f}-t_{0}$ is the total inference time.

The performance metrics are defined for comprehensive performance evaluation in Table 3.

The energy efficiency metric $\eta_{E}$ quantifies the trade-off between processing speed and power consumption, enabling direct comparison across heterogeneous platforms.

The cost efficiency $\eta_{C}$ is defined as the ratio of energy efficiency to hardware purchase cost, normalised to a $1000 reference cost so that the resulting value remains on a human-readable scale.

Hardware costs $C_{\mathrm{device}}$ correspond to the manufacturer in USD as listed in the supplier catalogue at the time of acquisition, shipping. Specifically: Dell G16 7630 = $1500; Dell XPS 13 9350 = $1200; NVIDIA Jetson Orin Nano Developer Kit = $499. Volume-discount pricing and used-market prices are not considered. The metric $\eta_{C}$ is intended as a one-shot purchase-decision indicator.

## 3. Experiments and Results

### 3.1. Benchmark Level-3

At this level, the items present more complex geometries and require grasping at specific, predefined points rather than at geometry-based or centroid locations. This makes the task the most challenging among the episodes. Our findings indicate that learning-based approaches are essential for addressing these difficulties, as the system can be trained to identify the most suitable grasping points for each object. Once these grasping points are determined, robot motion planning and control become critical to ensure that the manipulator can approach, grasp, detach, and deliver the item without collisions.

Using the proposed end-to-end system, the robot categorised the objects into two groups. (1) The conditioner and sweet-bag items, which have relatively simple geometric shapes, were handled using the traditional method. Although these items are deformable, they can still be grasped reliably with an appropriate grasping strategy. In this case, the gripper’s force control was tested on each item and tuned to ensure that it closed securely without causing damage. (2) The cleaner spray, Spam can, and tape, which require grasping at specific locations, were processed using the learning-based method. The performance of both approaches is summarised in Table 4.

**Figure 8:**
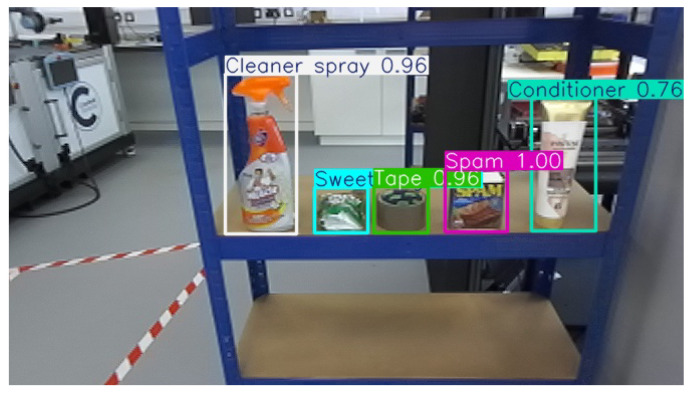
The initial detection module is responsible for classifying the object.

The first-stage detection results are illustrated in Figure 8. At this stage, the system performs object detection and classification, enabling the selection of the correct item from the order list. The consistently high confidence scores indicate that the model can accurately recognise and classify the items, providing a reliable foundation for subsequent grasp-point estimation and manipulation.

**Figure 9:**
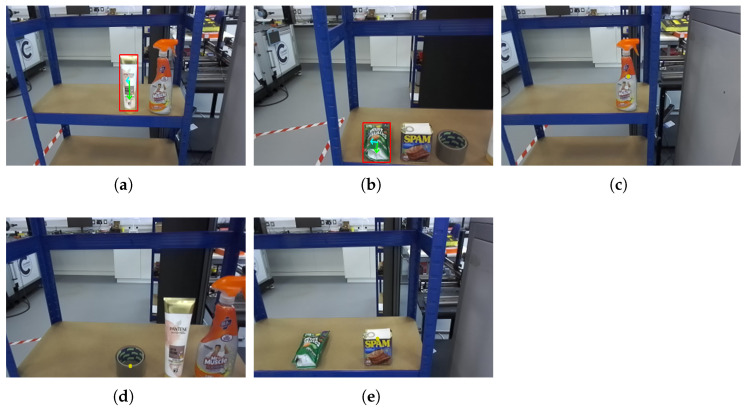
Grasp and pose estimation of each item: (**a**) The Conditioner is processed using the classical approach, where the red box denotes the bounding box from object detection, the yellow arrow represents the primary PCA axis used to determine orientation, and the blue arrow indicates the secondary axis; (**b**) the sweet bag using a classical approach as same as conditioner; (**c**) the cleaner spray using a learning approach; (**d**) the tape using a learning approach; (**e**) the spam using a learning approach.

The system then estimates the grasping point for each item using either the traditional method or the learning-based method, as illustrated in Figure 9. When the traditional approach is applied, the algorithm identifies the primary axis (yellow arrow) and secondary axis (blue arrow) of the object, and the intersection of these axes is selected as the grasping point. In contrast, the learning-based approach directly predicts an optimal grasping location, represented as a yellow dot, based on the model’s learned features.

The sweet bag recorded the lowest success rate at 60% (3/5), with both failures attributable to its deformable film structure and low-friction surface: once the gripper closed, the bag tended to slip or deform out of contact before the transport phase completed. Crucially, the grasp-point prediction and motion planning performed as intended in all five trials, the failures occurred at the physical grasping stage, not in perception or planning. Reliable pickup requires the gripper to fully close with enough force to pinch the film against its own weight; insufficient closing force allows the bag to slide out of the contact patch during lifting, while excessive force risks crushing the item. Tuning this closing force for deformable, low-friction items is a known limitation of position-controlled two-finger grippers and is noted as a future-work direction in Section 4.

The remaining four items were delivered reliably, yielding per-item execution times in the 6.9–8.3 s range. A consistent pattern emerges between the two grasp-planning strategies: the learning-based path (cleaner spray, Spam can, tape) completed each trial in roughly 7 s, while the classical segmentation-plus-PCA path (conditioner, sweet bag) required approximately 8 s. The gap reflects the additional cost of running FastSAM segmentation and PCA over the bounding-box region before a grasp point can be emitted, whereas the two-stage YOLO pipeline produces the grasp point directly.

Because the cycle-average power stays close to 17 W across all items and arm motion dominates the energy budget and is broadly similar between trials, CSR (Section 2.8) varies almost entirely with execution time. The Spam can, with the shortest average cycle at 6.89 s, therefore achieves the highest CSR (0.009), and the two classical-pipeline items the lowest (0.007). In these results, CSR functions as a compact efficiency indicator that combines computation and energy into a single number and confirms that the learning-based grasp path is the more efficient of the two sub-pipelines on this hardware. Further gains in CSR would come from reducing cycle time, for example, by compressing or fusing the detection and segmentation stages for classical-path items, rather than from reducing power, which is constrained by the robot arm rather than by the vision pipeline.

Success rate, however, depends on multiple factors beyond machine vision alone. Robot motion planning, grasp stability, and control precision all contribute significantly to overall performance. The results demonstrate that the proposed methodology and system design enable high success rates while effectively handling uncertainties and real-world conditions in the grocery pick-and-pack application.

Even though the Level 3 benchmark provides useful preliminary insight into system performance, the small number of trials per item (n=5) limits the statistical certainty of the observed success rates. To address this constraint, the Wilson 95% confidence interval offers a more reliable estimate of the underlying binomial proportion (success versus failure), particularly in small-sample settings [23].

(8) $$\hat{p}=\frac{x}{n},\;z=1.96$$

The Wilson interval is computed from the observed proportion $\hat{p}=x/n$, where *x* denotes the number of successful trials and *n* the total number of attempts, using the following formulation:

(9) $${\mathrm{CI}}_{\mathrm{Wilson}}=\frac{\hat{p}+\frac{z^{2}}{2n}}{1+\frac{z^{2}}{n}}\;\pm \;\frac{z}{1+\frac{z^{2}}{n}}\sqrt{\frac{\hat{p}\left(1-\hat{p}\right)}{n}+\frac{z^{2}}{4n^{2}}}$$

In Table 5, although several items achieved perfect observed performance (5/5), the corresponding Wilson 95% confidence intervals remain wide (56.6%, 100%), reflecting the statistical uncertainty inherent in small-sample evaluation rather than instability in the system itself. The sweet bag exhibits the lowest reliability (60% observed, CI 23.1%, 88.2%), consistent with its deformability and slippery surface, which introduce additional grasping difficulty. When aggregating all 25 trials, the system attains a more stable overall success rate of 92%, with a narrower Wilson interval of 75.1%, 97.8%, providing a more robust estimate of true performance across the benchmark.

To contextualise the performance of the proposed system, we compare it against GraspNet, a well-known real-time grasp detection method and one of the current state-of-the-art approaches [24]. GraspNet reports high grasp detection accuracy in the range of 87.3% to 90.5%, depending on the choice of α and β parameters, with a compact model size of 7.2 MB, a total memory footprint of 425 MB, and an inference speed of 133 ms on the NVIDIA Jetson TX1.

A direct comparison, however, is not possible due to differences in benchmark datasets, evaluation protocols, and computing platforms. In real-world experiments, GraspNet employs Faster R-CNN for object recognition and subsequently feeds the detected object into the GraspNet network for grasp estimation; thus, only the grasp detection stage is reported. In contrast, the system developed in this work performs end-to-end inference, encompassing both object detection and grasp estimation within a unified pipeline. Despite this broader scope, the overall model size (30.9 MB) remains smaller than that of GraspNet.

If the success rate is decomposed into grasp estimation, robot planning, and execution, all observed failures in our experiments stem from the execution stage, where objects slipped from the gripper due to surface properties or deformable shapes. The grasp detection component itself remains competitive, achieving accuracy above 90%. In terms of speed, GraspNet demonstrates excellent real-time performance at 133 ms on an edge device, whereas the proposed system currently averages 7.5 s (7500 ms) per full end-to-end cycle, approximately 56 times slower. It is important to note that this 7.5 s includes the entire perception-to-execution pipeline, not only grasp detection.

These observations highlight a clear direction for future work: improving system speed and incorporating architectural advantages from GraspNet-style lightweight grasp detection networks while maintaining the benefits of an integrated end-to-end design.

### 3.2. European Robotics League 2025

ERL 2025 provides an external validation of the system under real-world constraints, including unfamiliar item sets, public-venue WiFi, hardware damage, and time pressure, that are not replicable in a controlled laboratory. It demonstrates that the proposed system generalises beyond the benchmark item set and operates reliably without the controlled conditions of the laboratory evaluation.

**Figure 10:**
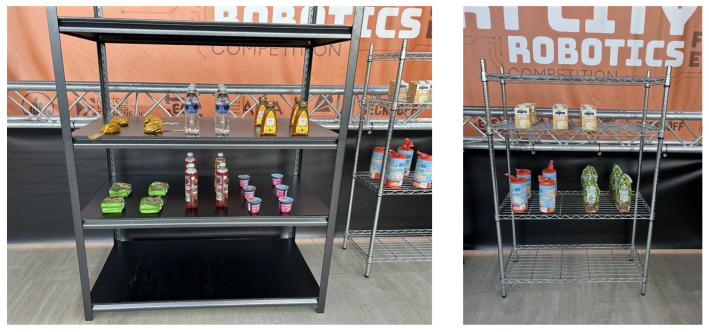
The set of objects arranged on the shelves.

In the competition, several challenging episodes are designed to represent key components of a smart city. Episode 4 focuses on the shopping pick-and-pack task, which addresses the problem of fulfilling customised orders. In this scenario, a user selects items from a predefined range of products on the shelves, such as a bag of chocolate coins, an Alpro custard carton, a bottle of honey, a jelly tart, a rice-cake pack, a bag of seed mix, a pasta pack, a bottle of water, and a Rubicon drink bottle as illustrated in Figure 10. An order-processing system then assigns the task of retrieving the requested item from the shop shelves and placing it into a delivery box.

**Figure 11:**
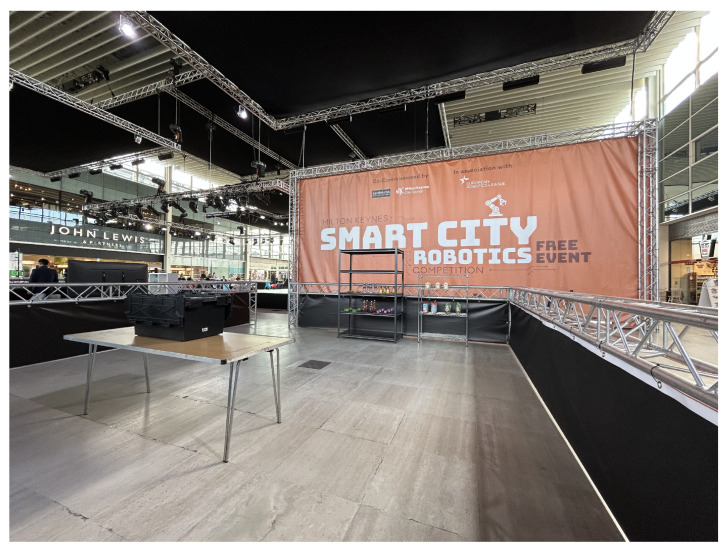
Layout of the competition area in episode 4 select and pack shopping.

The main scientific challenges in this episode involve the practical deployment of a mobile manipulator, the integration of machine vision techniques, and the execution of reliable robot control. These elements must work together to ensure that the robot can navigate the environment, perceive objects accurately, and perform grasping and placement actions robustly. The setup of the scenario is illustrated in Figure 11.

In terms of scoring, the competition includes both achievement points and penalties. A total of six points can be earned for successful execution of the task: (1) the robot announces the start of a new step; (2) the robot grasps a product; (3) the robot delivers the correct product to the designated delivery area; (4) all products in the order are successfully delivered; (5) the data hub correctly updates the remaining products on the shelves; and (6) the robot announces the end of the trial. The maximum score for this episode is 36 points. Penalties are applied for undesirable or unsafe behaviours. These include situations where the robot collides with furniture or objects; drops or damages an item; damages the environment; announces the completion of a step before the product is fully delivered; or announces the delivery of an item that is not part of the order or has already been delivered.

To achieve these criteria, the announcement and data-update requirements can be fulfilled through appropriate logical programming. Delivering the correct product is ensured by the object-detection module, which is trained on a dedicated dataset with sufficiently high confidence. The main challenges arise in grasping the product reliably, transporting it from the shelf to the delivery box without dropping it, and completing the full order, particularly because each item has a different and often challenging shape. These difficulties are compounded by the physical limitations of the robot, which uses a two-finger gripper with a maximum opening width of 8.5 cm. Additionally, avoiding collisions with furniture and surrounding objects is essential, as such incidents result in penalties and reduce the overall score.

**Figure 12:**
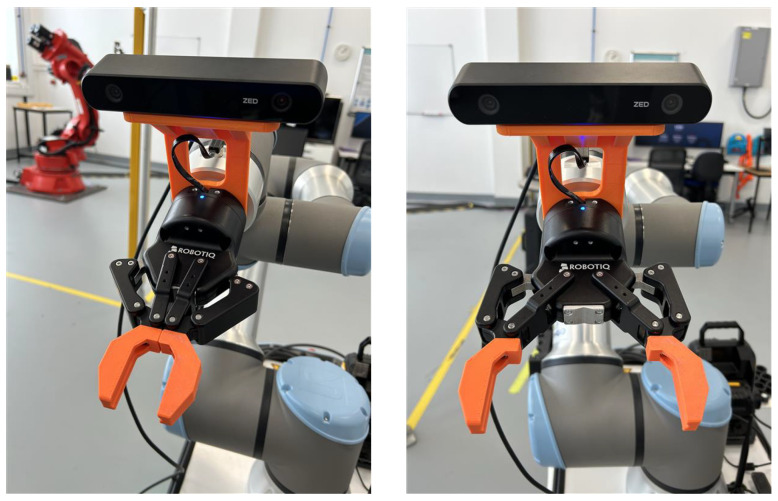
Design of the extended-width gripper using 3D printing for handling large items in ERL 2025.

According to our initial assessment, the widest item in the product set is the rice-cake pack, measuring nearly 12 cm. This exceeds the maximum opening width of the industrial Robotiq 2F-85 gripper installed on the manipulator, which is limited to 8.5 cm. To address this constraint, the gripper was modified to accommodate larger objects. The technical team designed a custom 3D-printed extension that increases the effective gripping width to approximately 15 cm, as illustrated in Figure 12. In addition, a roughened surface was applied to the fingertip regions to increase friction between the plastic gripper surfaces and the objects, thereby improving grasp stability.

In particular, the proposed end-to-end system was deployed to handle the competition task. Because the items used in this episode were new, a dedicated dataset had to be created. Images were captured from multiple distances and viewing angles to maximise variation. In total, 51 images were collected for training, and an 8-1-1 split was used for training, validation, and testing. Despite the small dataset, the model achieved effective detection performance, demonstrating that the system can operate reliably without requiring large-scale, high-quality datasets.

**Figure 13:**
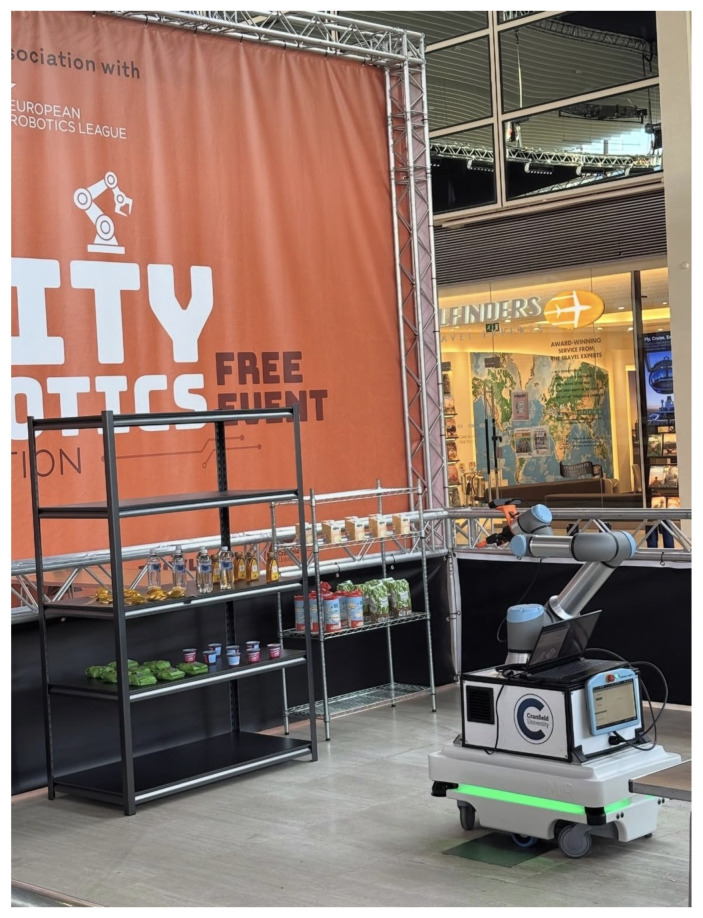
Execution of the System in the ERL 2025 Competition.

The system was also easily transferable to a laptop-based setup, which was necessary for on-site training and monitoring. During the competition, the public Wi-Fi connection was unstable, frequently disconnecting. To ensure reliable communication, a local network cable was used to connect directly to the MiR100 mobile base, enabling full robot control through its IP-based interface. This highlights not only the lightweight nature of the system but also its flexibility across different computing devices and network conditions. The operation of the system is illustrated in Figure 13.

**Figure 14:**
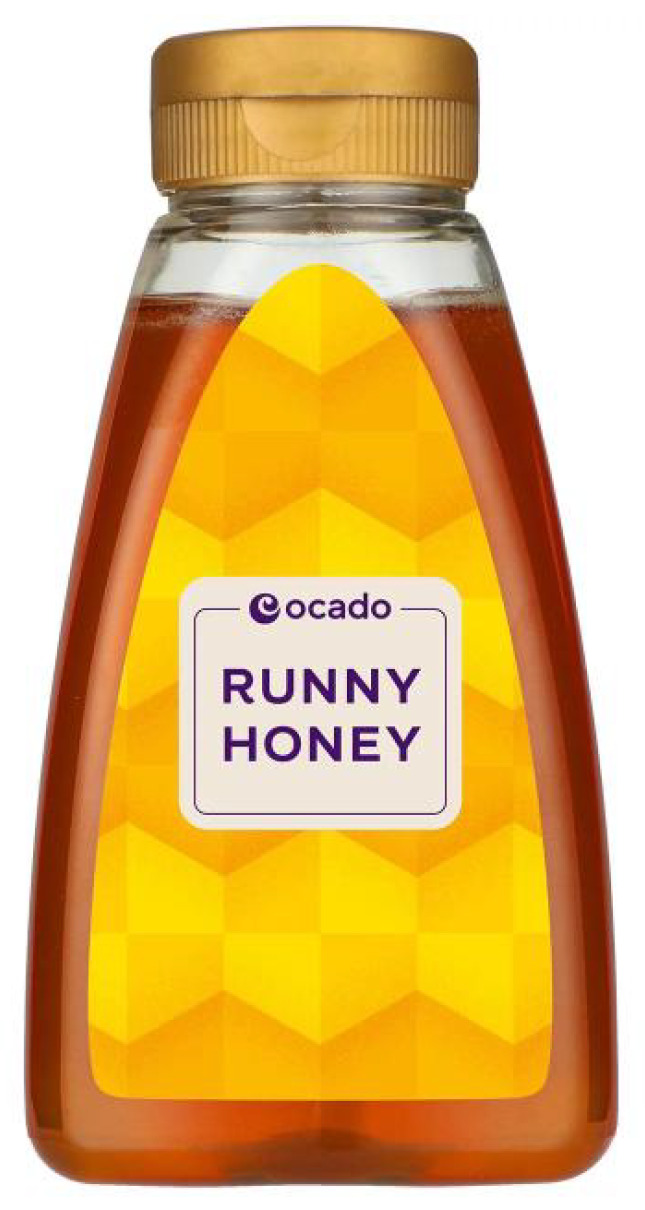
A bottle of honey.

As a result, the system successfully grasped three out of the six items: the jelly tart, the Rubicon drink bottle, and the bag of seed mix. The honey bottle was grasped successfully during some trials; however, in the final round the attempt failed due to its curved body shape, which caused the object to slip from the gripper after lifting, as illustrated in Figure 14. The rice-cake pack also presented difficulties. Because the shelf includes a stopper at the front edge, the base of the rice-cake pack collided with the stopper as the robot pulled it forward, causing the item to fall from the gripper. This incident was classified as a collision with furniture and resulted in a penalty.

**Figure 15:**
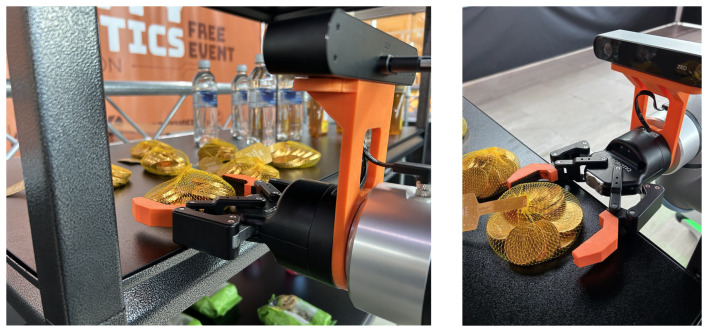
Challenges in Grasping a Bag of Chocolate Coins.

The chocolate-coin bag was not attempted during the competition because the physical configuration (deformable bag, flush with the shelf, requiring a pinch-style grasp) is outside the operational envelope of the Robotiq 2F-85 gripper and its 3D-printed extension (Figure 15). This is a system limitation rather than a strategic choice: even with unlimited attempts, the current hardware could not reliably grasp this item. Extending the hardware to handle such items is discussed in Section 4.

In total, the system achieved 17 out of 36 points, corresponding to three successfully delivered items with one penalty, and eventually the system was awarded first place in Episode 4 of ERL 2025 by the competition judges. We do not have access to the full per-team scoring breakdown for this episode, so direct numerical comparison with other participating teams is not possible in this paper; the placement itself is reported as an external, independently adjudicated validation of the system’s end-to-end performance under competition conditions.

However, an unexpected issue occurred because the same robot was also used in other competition episodes. During one of these tasks, the robot accidentally collided with the furniture, causing the camera holder to crack. This damage affected both the precision and accuracy of the vision system. When the robot returned to the shopping pick-and-pack task, rapid recalibration and interpolation were performed to restore functionality as much as possible. Nevertheless, the system could not be fully realigned, and there was insufficient time to fabricate a replacement camera mount. As a result, the overall performance in the final round was influenced by these hardware limitations.

### 3.3. Across Computation Platforms

To demonstrate the lightweight nature of the proposed system, we evaluated its performance on three different computing platforms: a Dell XPS laptop, a Dell G16 high-performance laptop, and an NVIDIA Jetson Orin Nano. The objective was to compare performance across devices with varying CPU capabilities and then assess the improvements gained when GPU acceleration was available. Cost and energy efficiency were also considered, as high-performance devices typically require greater financial investment and higher power consumption. For a fair comparison, all methods were executed under similar configurations on each device.

The system was implemented in Python and executed using Visual Studio Code 1.118.1, enabling consistent monitoring of inference results across platforms. Items from the benchmark Level-3 dataset were used to validate performance. Detection accuracy and grasp-point estimation remained consistent across all devices, confirming that the model behaves identically regardless of hardware. The primary differences were observed in processing speed, energy usage, and cost, which form the basis of the evaluation.

Experiments were conducted across four computing configurations representing different deployment scenarios, as detailed in Table 6. Each platform was evaluated in both CPU-only and GPU-accelerated modes where applicable.

The software stack consists of:

- PyTorch 2.0+ with CUDA 11.4/12.x for NVIDIA GPU acceleration;
- Intel Extension for PyTorch (IPEX) for Intel Arc GPU acceleration;
- Ultralytics YOLOv11 for object detection;
- FastSAM-s for instance segmentation;
- OpenCV 4.x for image processing and PCA computation.

Hardware monitoring utilizes platform-specific libraries:

- psutil: CPU metrics (utilisation, frequency, per-core stats, memory);
- pynvml: NVIDIA discrete GPU monitoring (power, utilisation, temperature, clocks);
- jetson-stats (jtop): Jetson platform monitoring via INA3221 sensors;
- wmi: Windows Management Instrumentation for Intel GPU (limited metrics).

From Table 7, objects processed through the part-detection pipeline (Cleaner Spray, Spam, Tape) achieve significantly faster inference times on the GPU (26–31 ms) compared with objects requiring full segmentation (226–230 ms). This 7.5 times difference is primarily attributable to the computational overhead introduced by FastSAM. On CPU platforms, the performance gap is less pronounced (1.2–1.5 times), indicating that detection time dominates the overall processing pipeline.

**Figure 16:**
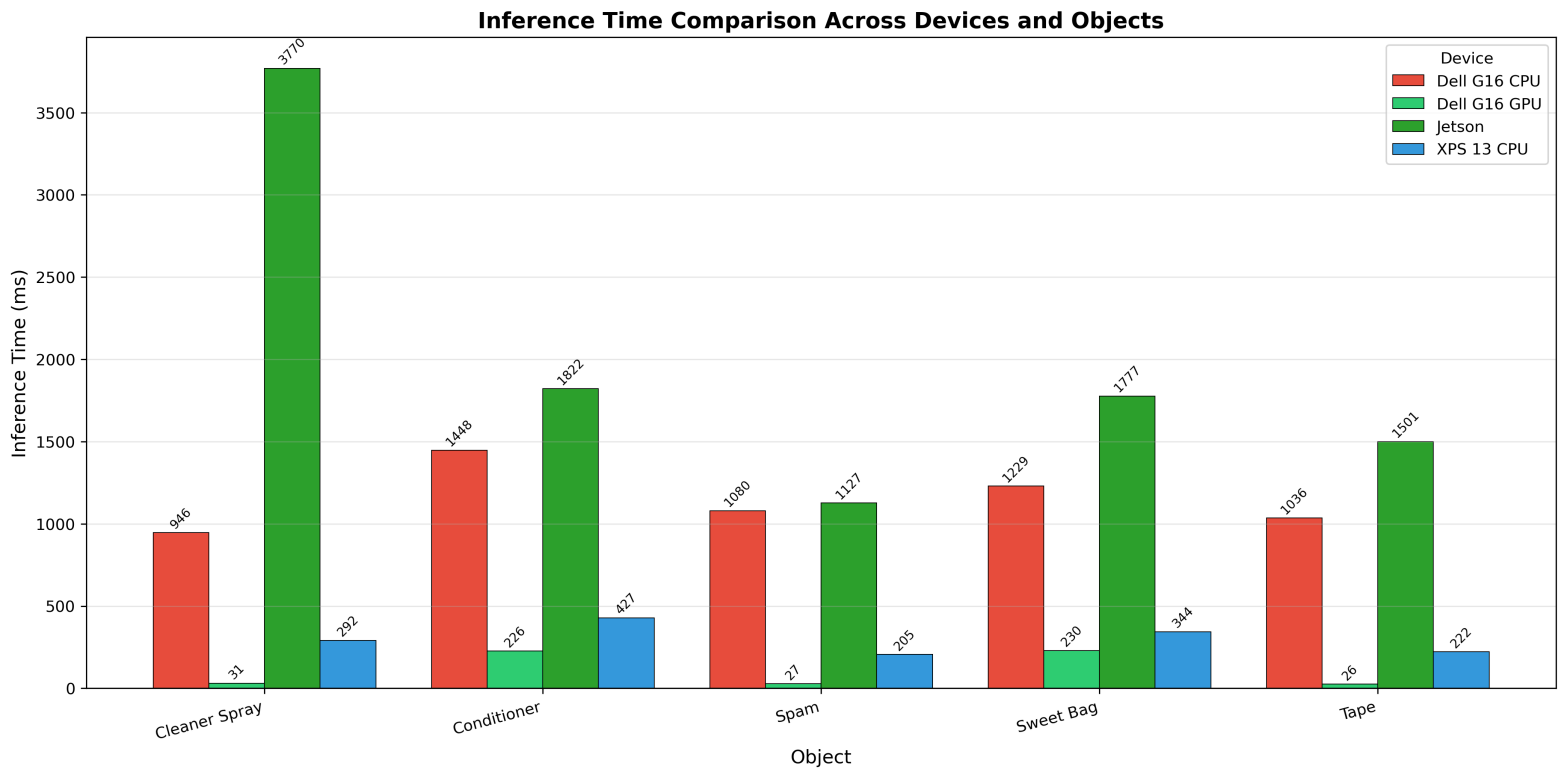
Inference time comparison across devices and object categories. Objects requiring segmentation (Conditioner, Sweet Bag) exhibit longer processing times than objects using part detection (Cleaner Spray, Spam, Tape).

The Jetson CPU exhibited anomalously slow performance for the Cleaner Spray (3769.80 ms), far exceeding the inference times of other objects. Further investigation revealed that this was caused by an extended part-detection search over a larger region of interest, resulting in substantially longer processing duration. In Figure 16, the bar chart highlights obvious differences in inference time across computing devices for all five items.

According to Table 8, the Jetson Orin Nano demonstrated the lowest absolute power consumption (6.56 W on average), consistent with its edge-computing design philosophy. However, when normalised by throughput, its energy consumption per frame is substantially higher than that of the GPU-accelerated configuration due to the extended processing time required for each inference.

**Figure 17:**
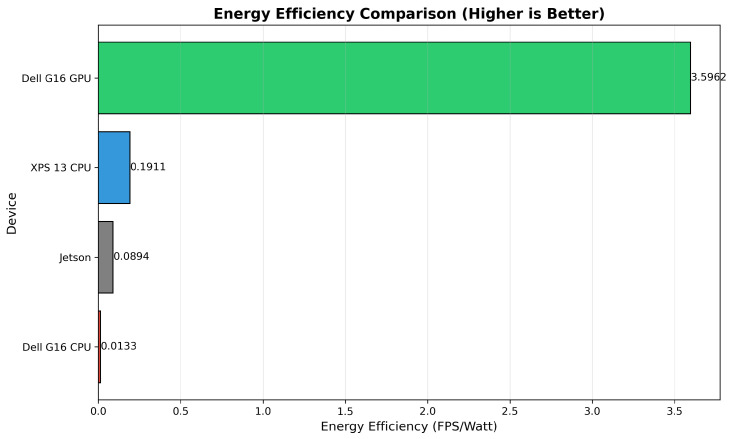
Energy efficiency comparison (FPS/Watt). Higher values indicate better efficiency. The Dell G16 GPU achieves 19 times higher efficiency than the next-best platform (XPS 13 CPU).

These results from Table 9 demonstrate that GPU acceleration delivers not only substantial performance gains but also significant energy savings, an increasingly critical consideration for sustainable and long-duration robotic applications. The relationship is clearly demonstrated in Figure 17, which highlights the differences in energy efficiency across devices.

Energy efficiency, defined as FPS per watt, provides one of the most comprehensive metrics for evaluating hardware suitability in edge-deployment scenarios. The Dell G16 GPU achieves an energy efficiency of 3.594 FPS/W, which is:

- 18.8× more efficient than XPS 13 CPU (0.191 FPS/W);
- 39.9× more efficient than Jetson CPU (0.090 FPS/W);
- 276.5× more efficient than Dell G16 CPU (0.013 FPS/W).

Regarding to Table 10, despite its higher purchase price, the Dell G16 GPU provides the best cost efficiency (2.396 FPS/W per $1000), outperforming the budget-oriented Jetson Orin Nano by a factor of 13.3. When operational costs such as electricity consumption are considered, the GPU’s advantage becomes even more pronounced, particularly for continuous or long-duration deployments.

**Figure 18:**
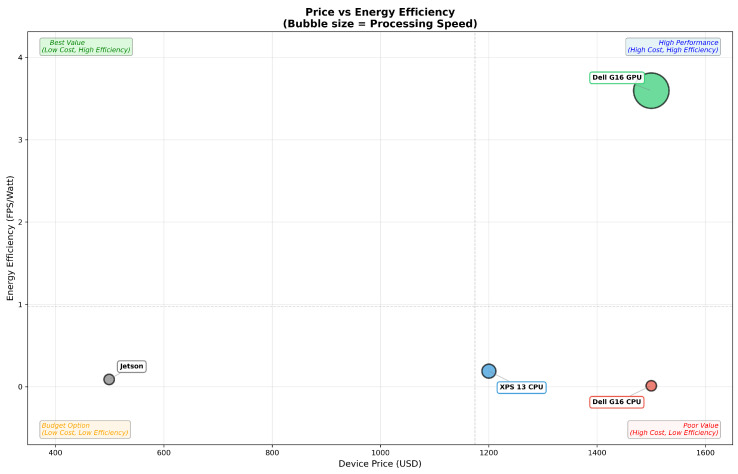
Price versus energy efficiency comparison. Bubble size is proportional to processing speed. The Dell G16 GPU offers the best overall value despite higher initial cost.

In CPU-only mode, the Jetson Orin Nano was the slowest device, particularly for more complex items such as the cleaner spray. However, for simpler objects like the Spam can, its inference time was comparable to that of the Dell G16 CPU. The Dell XPS 13 CPU demonstrated the highest efficiency, achieving inference speeds more than three times faster than the G16 CPU and over five times faster than the Jetson Orin Nano. When comparing speed (FPS) relative to energy consumption (W), the XPS 13 ranked highest, followed by the Jetson Orin Nano, with the G16 CPU performing the least efficiently. When cost was incorporated, evaluated as speed per watt per $1000, Figure 18 substantially shows that the Jetson Orin Nano offered the best value for money, with the XPS 13 s and the G16 last. When GPU acceleration was enabled, all devices showed substantial improvements in speed, energy efficiency, and cost effectiveness. This confirms that GPU support is highly beneficial for real-time robotic applications. Although GPUs increase hardware cost, the performance gains justify the investment when the project budget allows. Overall, the proposed method demonstrates strong compatibility across a wide range of computing devices. Its lightweight design results in a small model size and enables faster execution on more capable hardware, especially when GPUs are available. While budget considerations remain important, selecting hardware that aligns with the task requirements is even more critical. Modern computing platforms, from gaming laptops to compact ultrabooks and embedded AI devices, offer diverse options, and choosing the right specifications can significantly enhance system performance, operational efficiency, and cost effectiveness.

## 4. Discussion and Future Work

This research demonstrates a robust system architecture, effective algorithms across all modules, and lightweight computation validated through real-world scenarios, benchmark evaluations, and competition performance. Nevertheless, several areas remain open for improvement. For instance, the system has not yet been deployed in an actual grocery store environment with customers. In such settings, objects are often densely packed and cluttered on shelves, requiring more advanced approaching strategies and obstacle-avoidance behaviours to ensure safe and reliable manipulation.

Furthermore, while machine vision serves as the primary sensing modality, multimodal sensing is increasingly important. Sensor fusion can validate perception outputs and provide additional information to enhance reliability and task success. For instance, integrating force or tactile sensors into the gripper would allow the system to detect grasp failures. In the current system, task success is verified by a human observer at the delivery area, meaning the robot cannot autonomously recover from a failed grasp. With additional sensing, the robot could implement recovery behaviours, such as reattempting the grasp or readjusting its strategy.

Statistical scope and failure-mode attribution. The Level 3 benchmark in this paper uses 5 trials per item (25 in total), which is sufficient to characterise the system’s operating behaviour but does not support formal statistical inference on individual success rates. In particular, the 60% success rate observed for the sweet bag (3/5) reflects genuine execution difficulty associated with deformable, low-friction items. The present study does not decompose the two failure cases into vision-pipeline errors versus gripper or motion-execution errors, as per-stage logging was not instrumented in time for this evaluation.

A qualitative inspection of the sweet-bag failures indicates that the errors did not arise from grasp detection or motion planning. Instead, the failures occurred during execution due to insufficient grasp stability on a slippery, deformable surface. This behaviour is consistent with similar observations in ERL 2025, where the honey bottle, with a sloped neck and smooth plastic surface, frequently slipped from the gripper because the contact forces could not be applied perpendicular to the local surface normal. Such cases highlight the need for specialised techniques to handle deformable or low-friction objects. Future work should increase the trial count to n≥10 per item and incorporate per-stage failure logging so that vision, planning, and execution errors can be attributed separately. ERL 2025 provides complementary external validation under uncontrolled conditions, although with different items and without per-stage logging.

The sensing limitations of the ZED 2i stereo camera, including failures on reflective and transparent surfaces, are discussed in Section 2.2 as part of the sensing modality design rationale. Alternative depth cameras, such as the Intel RealSense D435 or D455, were considered during system design. Compared with the ZED 2i, these devices are lighter, cheaper (approximately USD 200–300 versus USD 450–500), and produce adequate depth maps for close-range tabletop manipulation. However, the ZED 2i was selected [25] because (a) it provides a larger baseline stereo geometry that improves accuracy at the 0.5–3.0 m object distances relevant to shelf picking, (b) it supports the low-resolution-with-padding detection strategy described in Section 2.2, and (c) its SDK provides direct integration with ROS and Python, accelerating development. For cost- or weight-constrained deployments, the Intel RealSense D435i would be a suitable alternative and is expected to be compatible with the pipeline with minor calibration adjustments.

The Robotiq 2F-85 gripper, even with the 3D-printed extension, was insufficient for two items in the ERL 2025 set: the chocolate-coin bag (deformable, flush with the shelf surface, requiring a pinch grasp the 2F-85 cannot perform) and the rice-cake pack (depth of 12 cm exceeds the unmodified finger reach). These cases illustrate a fundamental limitation of two-finger parallel grippers: they excel at grasping objects with two parallel contact faces but fail on items that require enveloping, pinching thin flexible material, or applying non-planar contact forces. Bio-inspired grippers (e.g., soft pneumatic fingers) and dexterous multi-finger hands (e.g., Allegro, Shadow) can address some of these failure cases but introduce additional control complexity, higher cost, and in some cases higher weight, trade-offs that must be carefully evaluated against the deployment constraints of a mobile platform.

From an algorithmic perspective, this work analyses how grasping points can be extracted directly from RGB images while considering grasp stability and grasp affordance. By focusing only on essential grasping points, the system avoids redundant computation. In contrast, other approaches often generate a large number of candidate grasp points and then regress them to the most feasible option for the robot. While this can increase opportunities for successful grasps in certain scenarios, it comes at the cost of higher computation and slower processing. Our approach prioritises efficiency by identifying only the most meaningful grasp-affordance regions.

To further enhance system performance, several algorithmic directions can be explored. Instance segmentation, for example, should incorporate classification capabilities so that precise object information can be extracted without relying on redundant pipelines such as running object detection and segmentation simultaneously. Bounding boxes from object detection frequently fail to represent object shapes accurately, whereas instance segmentation provides pixel-level precision. Moreover, if future models can estimate the 3D geometry of objects, they would enable full six-degree-of-freedom pose estimation, resulting in more accurate robot motion planning and approach strategies.

In addition, grasp-estimation networks should be developed with a focus on low computational cost and efficiency. In this work, YOLOv11 demonstrated exceptional performance in identifying grasping points with a nano model, outperforming existing grasp-estimation networks that are often complex to train and still struggle to achieve high accuracy. We have attempted to implement a grasp-estimation network based on the current state-of-the-art method, GraspNet. However, our experiments showed that using grayscale images did not reduce computational load compared to RGB inputs, despite reducing the number of channels from three to one. Hyperparameter tuning provided moderate improvements, but the resulting model still could not match the Level-3 benchmark performance achieved by YOLOv11. These findings indicate that current grasp-estimation networks require further refinement and task-specific optimisation to be competitive in practical, real-time applications.

## 5. Conclusions

In the coming years, society is moving toward the vision of a smart city, where robots and AI technologies enhance daily life in positive and practical ways. Grocery shopping is one domain in which a mobile manipulator can deliver a fully autonomous pick-and-pack solution. In this research, a complete system is proposed, integrating an end-to-end computational grasping pipeline, robot motion planning and control, and a graphical user interface. The system was validated using the grocery pick-and-pack benchmark and further demonstrated in the European Robotics League 2025 competition. Cross-platform evaluation was also conducted to assess performance on different computing devices.

The results show that the system achieved a high average success rate of 92% across five items on the Level-3 benchmark and an average picking speed of 7.5 s on an edge-computing device. The system also won the competition with a score of 17 out of 36 points. Furthermore, the lightweight design enables a compact model size of only 30.9 MB and seamless transferability across hardware platforms. The experiments also indicate that GPU acceleration can significantly improve speed and energy efficiency, although this comes with additional cost. The choice ultimately depends on budget and application requirements, but even an affordable GPU can provide meaningful benefits.

Overall, this project aims to contribute an essential concept toward shaping the future of smart-city autonomy, supporting and enhancing human living in a constructive and sustainable direction. Future work is expected to involve deploying such systems in real-world grocery stores, where they can collect large-scale, real-world data to identify remaining challenges. This will enable further refinement and development of the system to ensure effective, practical, and reliable operation in everyday environments.

## Figures and Tables

**Table 1:** Key technical differences between this work and the authors’ prior paper [4].

Aspect	Prior Work [4]	This Paper
Application	Geometric grocery items	Full option for general grocery items and GUI
Benchmark level	Levels 1–2 (geometric)	Level-3 (deformable + non-centroid)
Grasp-point method	PCA only	PCA + learning-based (2nd stage detection)
Evaluation venue	Laboratory	Laboratory + ERL 2025 live competition
Cross-platform study	Single device	Four hardware configurations
Energy/cost metrics	CSR only	CSR, $\eta_{E}$, $\eta_{C}$ defined and compared

**Table 2:** Power measurement methods by platform.

Platform	Method	Library	Measurement Type
Dell G16 (CPU)	Utilisation-based estimation	psutil	Estimated
Dell G16 (GPU)	NVML sensor reading	pynvml	Direct
Dell XPS 13 (CPU)	Utilisation-based estimation	psutil	Estimated
Jetson	INA3221 sensor reading	jtop	Direct

**Table 3:** Performance metrics definition.

Metric	Formula	Unit
Image Load Time	$T_{\mathrm{load}}$	ms
Detection Time	$T_{\det}$	ms
Segmentation Time	$T_{\mathrm{seg}}$ (includes PCA)	ms
Total Inference Time	$T_{\mathrm{total}}=T_{\mathrm{load}}+T_{\det}+T_{\mathrm{seg}}$	ms
Throughput	$\mathrm{FPS}=\frac{1}{T_{\mathrm{total}}}$	frames/s
Average Power	$\bar{P}=\frac{1}{N}\sum_{i=1}^{N}P_{i}$	W
Energy per Frame	$E_{f}=\bar{P}\cdot T_{\mathrm{total}}$	J
Energy Efficiency	$\eta_{E}=\frac{\mathrm{FPS}}{\bar{P}}$	FPS/W
Cost Efficiency	$\eta_{C}=\frac{\eta_{E}}{C_{\mathrm{device}}}\times 1000$	$\frac{\mathrm{FPS}/W}{\$1000}$

**Table 4:** Result on Level-3 benchmark.

Grocery Items	Weight (g)	Execution Times (s)	Average Time (s)	Success Rate	Average Energy Consumption (W)	CSR
Conditioner	295	8.13 8.63 8.19 8.28 8.19	8.284	100% (5/5)	17	0.007
Sweet bag	147	8.35 8.32 8.25 8.28 8.29	8.298	60% (3/5)	17	0.007
Cleaner spray	481	6.96 6.97 7.29 7.02 7.01	7.050	100% (5/5)	17	0.008
Spam	407	6.83 6.91 6.70 7.12 6.90	6.892	100% (5/5)	17	0.009
Tape	82	6.71 6.79 6.83 7.47 6.97	6.954	100% (5/5)	17	0.008

**Table 5:** Result on Level 3 benchmark using Wilson 95% confidence intervals.

Grocery Items	x/n	Point (%)	Wilson 95% CI
Conditioner	5/5	100	56.6%, 100.0%
Sweet bag	3/5	60	23.1%, 88.2%
Cleaner spray	5/5	100	56.6%, 100.0%
Spam	5/5	100	56.6%, 100.0%
Tape	5/5	100	56.6%, 100.0%
All items	23/25	92	75.1%, 97.8%

**Table 6:** Hardware specifications of evaluation platforms.

Platform	Processor/GPU	TDP (W)	Memory	Price (USD)
Dell G16 7630	Intel i7-13650HX (14C/20T) NVIDIA RTX 4060 (3072 CUDA)	55–157 140	32 GB 8 GB VRAM	$1500
Dell XPS 13 9350	Intel Core Ultra 5 226V (8C/8T) Intel Arc Graphics (integrated)	17–28	16 GB	$1200
Jetson Orin Nano	ARM Cortex-A78AE (6C) NVIDIA Ampere GPU (1024 CUDA)	7–15	8 GB	$499

**Table 7:** Object-specific inference times (ms).

Object	Pipeline	G16 CPU	G16 GPU	Jetson	XPS 13
Two-stage detection pipeline (Detection → Part Detection):					
Cleaner Spray	Part Det.	945.76	31.01	3769.80	291.68
Spam	Part Det.	1080.13	27.46	1126.77	205.39
Tape	Part Det.	1035.68	26.00	1500.58	221.59
Classical pipeline (Detection → FastSAM → PCA):					
Conditioner	Segment.	1447.81	225.94	1821.53	427.49
Sweet Bag	Segment.	1229.13	229.71	1776.76	344.08

**Table 8:** Estimated power consumption and energy results.

Platform	Avg. Power (W)	Max Power (W)	Energy/Frame (J)	Measurement
Dell G16 GPU	6.45	11.07	0.66	Direct (NVML)
Dell G16 CPU	66.99	157.00	76.72	Estimated
Jetson	6.56	7.51	13.12	Direct (INA3221)
XPS 13 CPU	18.90	28.00	5.63	Estimated

**Table 9:** Estimated energy efficiency comparison.

Platform	FPS	Power (W)	$\eta_{E}$ (FPS/W)	Relative	Rank
Dell G16 GPU	23.18	6.45	3.594	1.00×	1
XPS 13 CPU	3.61	18.90	0.191	0.053×	2
Jetson	0.59	6.56	0.090	0.025×	3
Dell G16 CPU	0.89	66.99	0.013	0.004×	4

**Table 10:** Cost-effectiveness analysis.

Platform	Price (USD)	$\eta_{E}$ (FPS/W)	$\eta_{C}$ (FPS/W/$1000)	Rank
Dell G16 GPU	$1500	3.594	2.396	1
Jetson	$499	0.090	0.180	2
XPS 13 CPU	$1200	0.191	0.159	3
Dell G16 CPU	$1500	0.013	0.009	4

## Data Availability

The original contributions presented in this study are included in the article; further inquiries can be directed to the corresponding author.
